# Temperature Dependence of Poly(3-hydroxybutyrate-*co*-3-hydroxyhexanoate) Biodegradation in Agricultural Soils

**DOI:** 10.1021/acs.est.5c08707

**Published:** 2026-02-23

**Authors:** Juliana R. Laszakovits, Silvan Arn, Ralf Kägi, Silvan Liechti, Flora Wille, Kristopher McNeill, Michael Sander

**Affiliations:** † Institute of Biogeochemistry and Pollutant Dynamics (IBP), Department of Environmental Systems Science, 27219 ETH Zuerich, Zuerich 8092, Switzerland; ‡ Eawag, Swiss Federal Institute of Aquatic Science and Technology, Ueberlandstrasse 133, Duebendorf CH-8600, Switzerland

**Keywords:** polyhydroxyalkanoates, biodegradation, soil, temperature, biodegradable polymers, biodegradation rates

## Abstract

Biodegradable polyesters are increasingly used in agricultural applications with soil biodegradation as the targeted postapplication end-of-life. Soil biodegradability is typically established in laboratory soil incubations at constant temperatures exceeding those in many field soils, highlighting the need for robust temperature-biodegradability relationships to transfer laboratory-determined biodegradation rates to field conditions. This work systematically assesses the temperature dependence of the biodegradation of three poly­(3-hydroxybutyrate-*co*-3-hydroxyhexanoate) (PHBHHx) variants, containing differing molar amounts of 3-hydroxyhexanoate (where x = 5, 9, and 12%) in three agricultural soils. Scanning electron microscopy images of PHBHH9 films incubated for 18 days showed increasing film surface colonization by fungi and/or filamentous bacteria from 5 to 15, 25, and 35 °C, with dense hyphal networks and film disintegration at the higher tested temperatures. Soil biodegradation of PHBHHx powders over the same temperature range was followed by extracting and quantifying residual PHBHHx mass over time by proton nuclear magnetic resonance spectroscopy. Fitting of these residual masses with a shoulder-log linear kinetic model yielded PHBHHx-variant-independent initial lag phases and biodegradation rate constants. Increasing the temperature shortened the initial lag phase and increased the subsequent rate of PHBHHx biodegradation. Arrhenius rate law analysis revealed soil-specific activation energies that correspond to approximately 2- to 5-fold changes in biodegradation rates per 10 °C. This work establishes temperature as a key abiotic factor controlling polyester biodegradation rates in soils and guides more accurate extrapolation of laboratory-determined biodegradation rates at elevated temperatures to field conditions.

## Graphical abstract

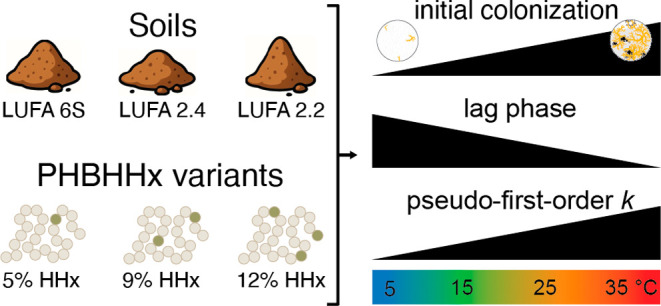

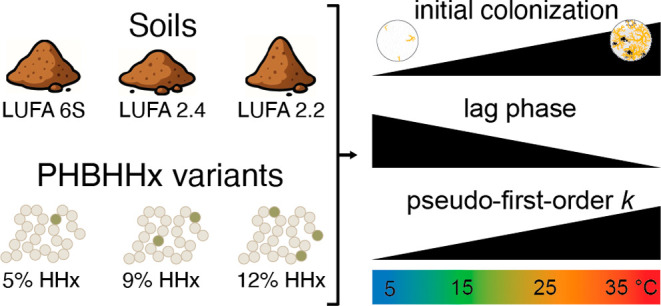

## Introduction

The use of biodegradable polyesters in specific applications is increasingly recognized as one tool in a range of solutions to overcome the release of persistent plastics to the environment.^1,2^ This is particularly true for applications in which plastics are directly used in the open environment and when complete collection of the plastics after use is impossible.^1,2^ Many agricultural plastic products, including thin mulch films and seed coatings, fall into this category and, when these products contain and release conventional polymers, result in their long-term accumulation in agricultural soils.^1–3^ Plastics composed of biodegradable polyesters are instead designed to undergo complete metabolic utilization by soil microorganisms and, thereby, prevent plastic accumulation in soils.^1^

The soil biodegradability of polymers for such applications is tested in and certified based on laboratory soil incubations coupled to respirometric analysis of polymer carbon conversion to CO_2_.^4,5^ These soil incubations are typically conducted at a constant temperature between 20 and 28 °C, commonly 25 °C.^4,5^ This temperature is at the upper end of the range of temperatures of temperate soils under *in situ* conditions in field environments, with mean annual temperatures in temperate zone top soil ranging from around 0 to 20 °C.^6^ Because higher temperatures are expected to favor biodegradation, concerns have been raised regarding the transferability of laboratory-determined polymer biodegradation rates and extents to *in situ* conditions in the field with often lower and highly variable temperatures.^7,8^ Assessing the transferability of laboratory data to the field requires systematically studying the effect of temperature on polymer biodegradation dynamics in soils.

Increasing temperature over environmentally relevant temperature ranges is expected to increase the activity of psychrophilic and mesophilic soil microorganisms involved in biodegradation, provided that no other limitations arise (e.g., soil drying) and that temperature remains below threshold values above which it negatively impacts the microbiota.^9,10^ With increasing temperature, the rate of initial colonization of the polyester surfaces by soil microbial degraders, the secretion levels and activity of extracellular microbial esterases that catalyze the hydrolysis of polyesters into small oligomers and monomers,^7,11^ as well as the rate at which these products are taken up and metabolically converted by soil microorganisms^12–18^ are all expected to increase. In addition, increasing temperature can induce changes in the physical properties of polyesters that alter their biodegradability.^9,13,19–21^ For instance, as incubation temperatures approach the melting temperature of crystalline spherulites in semicrystalline polyesters, their enzymatic hydrolyzability substantially increases.^22–24^

A viable approach to systematically study the temperature dependence of polyester surface colonization and biodegradation is to conduct laboratory soil incubations over a range of environmentally relevant temperatures while monitoring both microbial growth on the polyester surface and polyester mass loss. Polyhydroxyalkanoates (PHAs), a class of biobased, microbially derived, and biodegradable polyesters,^25,26^ are promising candidates in such assessments for three main reasons. First, many PHAs readily biodegrade in a range of receiving environments, including soils.^27–29^ High biodegradability enables following PHA soil biodegradation rates even at low incubation temperatures over reasonable incubation times. Second, one PHA, poly-3-hydroxybutyrate, is accepted as a positive control in soil biodegradation tests for the certification of polymer biodegradability.^5^ Third, information on the temperature dependence of PHA biodegradation in soils may also inform the temperature dependencies of other commercially important biodegradable polymers that are also polyesters (e.g., polybutylene adipate-*co*-terephthalate, polybutylene succinate, and polylactic acid). A major PHA class is poly­(3-hydroxybutyrate-*co*-3-hydroxyhexanoate) (PHBHHx, where x refers to the 3-HH content), which contains the two monomeric units 3-hydroxybutyrate (3-HB) and 3-hydroxyhexanoate (3-HH) at varying relative ratios and is already used in commercial products. Variations in the comonomer ratio in PHBHHx alter their physicochemical properties^7,28,30,31^ and may alter their biodegradability.^26,28,29,32^ Establishing the effect of PHA structure on biodegradability could inform the design of PHA with tunable biodegradability characteristics.

Here, we systematically studied the effect of incubation temperature on the biodegradation dynamics of three commercially relevant PHBHHx variants with 3-HH contents of 5, 9, and 12 mol % (hereafter termed PHBHH5, PHBHH9, and PHBHH12, respectively) in three standard agricultural soils, LUFA 6S, 2.4, and 2.2. The three soils were selected to cover a range of soil types and properties. Two sets of incubation experiments were conducted. First, we assessed the effect of temperature on the initial dynamics of microbial film colonization by incubating solvent-cast films of PHBHH9 in the three soils at 5, 15, 25, and 35 °C for 18 days, followed by retrieval and imaging of the films using scanning electron microscopy (SEM) and subsequent image analysis to quantify the extent of microbial surface colonization and film disintegration. Second, we followed the biodegradation of the three PHBHHx variants as powder added to the three soils at temperatures ranging from 5 to 35 °C. Biodegradation was monitored by determining the residual PHBHHx mass in soil over the course of the incubation using Soxhlet solvent extraction of residual PHBHHx from soil followed by its quantification by proton nuclear magnetic resonance (^1^H NMR) spectroscopy.^15,33,34^ The residual PHBHHx mass data was fitted by a shoulder-log linear biodegradation model to quantify both the lengths of the initial lag phases and the subsequent pseudo-first order rate constants of the PHBHHx biodegradation. We investigated the effects of the temperature, soil, and 3-HH content on PHBHHx biodegradation. Finally, we assessed to what extent the temperature dependence in pseudo-first order biodegradation rate constants was described by the Arrhenius rate law and determined apparent activation energies (*E*_a_) for biodegradation. The temperature dependence of PHBHHx biodegradation in soils could not only inform the transferability of biodegradation rates determined in laboratory soil incubations at elevated temperatures to the field with lower and variable temperatures but also help interpret the biodegradation dynamics of polyesters in field soils under varying temperatures.

## Materials and Methods

### Polymers

Three PHBHHx variants (PHBHH5, PHBHH9, and PHBHH12) were provided as powders from Kaneka Corporation (Belgium) and used as received. The 3-HH contents of 5, 9, and 12 mol % (of total molar monomeric units) were determined by ^1^H NMR (Figures S1–S5 and Table S1).

### Soils

Three agricultural standard soils were obtained from LUFA-Speyer in July 2021: LUFA 2.2 (soil type according to the USDA classification: sandy loam), 2.4 (loam), and 6S (clay). Key soil physicochemical properties are listed in Table S2.

### Chemicals

Chloroform (LC grade), trichloroethylene (TCE; ACS reagent grade), glutaraldehyde (50% w/w), hexamethyldisilazane (HMDS; synthesis grade), and 1X phosphate-buffered saline (PBS) were obtained from Sigma-Aldrich. Ethanol (denatured with IPA) was from Alcosuisse, methanol (LC grade) from Fisher, deuterated chloroform (99.8% D) from Eurisotop, and 1,4-dimethoxybenzene (DMB; 99+%) from Acros Organics.

### Soil Incubations of Films to Assess Microbial Colonization

#### Preparation of PHBHH9 Films

PHBHH9 was dissolved in a chloroform:TCE (9:1) mixture to a concentration of 1.5% PHBHH9 by mass. Solution aliquots (11 mL) were transferred into glass Petri dishes (diameter: 9.1 cm, area: 65 cm^2^) and the solvents were left to evaporate overnight at room temperature, resulting in solvent-cast PHBHH9 films (estimated thicknesses of ∼13 μm based on geometry, volume, and concentration used). Circular film pieces (diameter: 1.1 cm) were cut out of the larger cast film using a hole punch die and subsequently stored in a desiccator until use.

#### Preparation of Soils

Each soil was first adjusted to 50% of its maximum water-holding capacity and subsequently preincubated in aluminum containers at 5, 15, 25, and 35 °C for at least 2 weeks to allow for microbial adaptation to the incubation temperature prior to use for PHBHHx incubations.

#### Scanning Electron Microscopy Imaging of Microbial Colonization

For each soil and temperature, two PHBHH9 films were vertically placed at two different locations in the soils and incubated for 18 days. Following retrieval from the soils, the films were prepared for SEM imaging by first gently rinsing them with 1X PBS (to remove loosely attached soil particles), followed by submerging them in 2.5% glutaraldehyde in 1X PBS (v/v) for 30 min, rinsing them in 1X PBS for 20 min, and dehydrating them by placing them into a 70% ethanolic solution and subsequently pure ethanol, each for 1 h.^17^ Finally, the films were dried by submerging them in HMDS, followed by slow evaporation of the HMDS overnight.^35^ The films were then mounted on SEM stubs and coated with platinum (4 nm) using an EM ACE600 sputter coater (Leica Microsystems).^17^ The films were imaged by SEM (Gemini460, Zeiss, Germany) operated at an acceleration voltage of 5 kV and a current of 200 pA using ATLAS software to automate the imaging process. Secondary electrons (SE) detected by either an Everhart-Thornley-type detector or an InLens detector were used for image formation. The generated images were analyzed by ImageJ and Python to quantify the total length of individual hyphae on the films and areas of both dense colonies and holes in the films. The remaining area was categorized as an intact film. Full details on the image analysis are presented in Text S1 and Figure S6.

#### Soil Incubations to Determine PHBHHx Biodegradation

Mass loss of PHBHHx by Soxhlet extraction and ^1^H NMR was chosen over respirometry to allow for a higher sample number and to directly quantify residual PHBHHx. This approach overcomes potential artifacts and interpretational ambiguities resulting from respirometry (e.g., potential priming effects as well as underestimation of biodegradation in system where a substantial fraction of polymer carbon is used for microbial biomass formation).^36^ Soils were prepared as described above. Incubations were carried out in 50 mL centrifuge tubes containing ∼20 g of wet soil and ∼20 mg of powder of a given PHBHHx variant covered with punctured caps (to allow gas exchange) in temperature-controlled incubators (set for all soils to 5, 15, 25, and 35 °C and, for LUFA 6S, additionally to 20 and 30 °C) at 100% relative humidity. Exact masses of soil and PHBHHx for each incubation tube were gravimetrically recorded (uncertainty of the balance: 0.1 mg). Triplicate incubation samples were prepared for each sampling time point and treatment (i.e., soil, temperature, and PHBHHx variant). At different incubation times, triplicate samples were removed from the incubators and frozen at −20 °C. The samples were then freeze-dried and sealed until extraction. To this end, the samples were transferred into cellulose extraction thimbles (22 mm × 80 mm, VWR), followed by Soxhlet-extracting the residual PHBH on a six-position extractor (Behr Labor Technik, Germany; Model: S 306) for 1 h using 70 mL of a boiling 9:1 (v:v) chloroform:methanol cosolvent mixture at 80% power (approximately 26 cycles).^33^ The extract containing residual PHBHHx was dried by evaporating the solvent, followed by reconstituting the residual PHBHHx in deuterated chloroform with 2 mg mL^–1^ DMB as an internal standard. The mass of residual PHBHHx was quantified using quantitative proton nuclear magnetic resonance spectroscopy.^33,34^ Quality control spike recovery experiments were performed and showed 97 ± 4% recovery of the three PHBHHx variants from the three tested soils (Figure S7). The spectra were collected on a 400 MHz Bruker Ascend NMR spectrometer (number of scans: 128; pulse width: 14 μs with a 15 s delay between pulses and 16 dummy scans). All data were processed in Mestrenova (Mestrelab research).^37^ Example NMR spectra and details on the data processing and calculations of the residual PHBHHx mass are provided in Figures S1–S3 and Text S2, respectively. The residual PHBHHx mass of a given sample was normalized to the initial PHBHHx mass added to that sample and expressed in percent. Residual PHBHHx is reported as the average of triplicate incubations with error bars corresponding to standard deviations between the triplicates.

#### Mathematical Description of Biodegradation Data

The residual PHBHHx mass data plotted versus incubation time was fitted by a shoulder-log linear kinetic model, which considers an initial lag phase followed by exponential degradation (eq 1).

$$m_{\mathrm{PHBHHx}}(t)=m_{\mathrm{PHBHHx}}(t_{0})\cdot e^{-k\cdot t}\cdot \frac{e^{k\cdot L}}{{1+(e}^{k\cdot L}{-1)e}^{-k\cdot t}}$$ 1

where *m*_PHBHHx_(*t*) and *m*_PHBHHx_(*t*_0_) (both in mg) are the PHBHHx masses remaining at incubation time *t* (d) and initially added to the same incubation vial, respectively, *L* (d) is the duration of a lag phase starting at the onset of the incubation, and *k* (d^–1^) is the pseudo-first order mass-loss rate constant.^38^ When *L* = 0, the model converges to the commonly used pseudo-first order kinetic degradation model.^10,39^ For each model, we assessed whether *L* was a statistically significant variable in the shoulder-log linear model, as well as comparing the Akaike information criterion (AIC), the Bayesian information criterion (BIC), chi-squared (χ^2^), and −2log­(likelihood) between the shoulder-log linear model when *L* is fit freely (Table S3) and when *L* is 0 (Table S4). When *L* was not statistically significant (*p*-value > 0.05), we chose to set *L* = 0. The model was fit using the average of three replicates for each time point as the inclusion of the individual replicates would invalidate the underlying assumptions of the shoulder-log linear model, which assumes each data point is completely unique. Differences in the *L* and *k* parameters quantitatively capture temperature effects on PHBHHx biodegradation in soil.

## Results and Discussion

### Initial Microbial Colonization and Disintegration of PHBHH9 Films

#### Qualitative Image Analysis

Large sections of the PHBHH9 films, after their incubation in the three soils for 18 days at 5, 15, 25, and 35 °C, were imaged by SEM, resulting in 5.8 mm × 5.8 mm to 8.2 mm × 8.2 mm stitched mosaic images (Figures S8–S10). Examples of individual SEM images of PHBHH9 films incubated in LUFA 6S soil are shown in Figure 1 (analogous example images for LUFA 2.4 and 2.2 soils are shown in Figures S11–S12, respectively). The images show increasing extents of surface colonization by hyphal (i.e., filamentous) structures with increasing incubation temperature from 5 to 35 °C. At 5 °C, only a few hyphae were found on the film surface, which also was mostly intact with no or only little disintegration (Figure 1A). Hyphal structures were more abundant at 15 °C with signs of PHBHH9 surface erosion along the hyphae (Figure 1B). At 25 °C and particularly at 35 °C, the hyphal network on the surface was extensive, with parts of the film surface so densely colonized that individual hyphae were no longer distinguishable (Figure 1C and D). Disintegration was extensive at these higher temperatures with holes forming in the PHBHH9 film.

**Figure 1:**
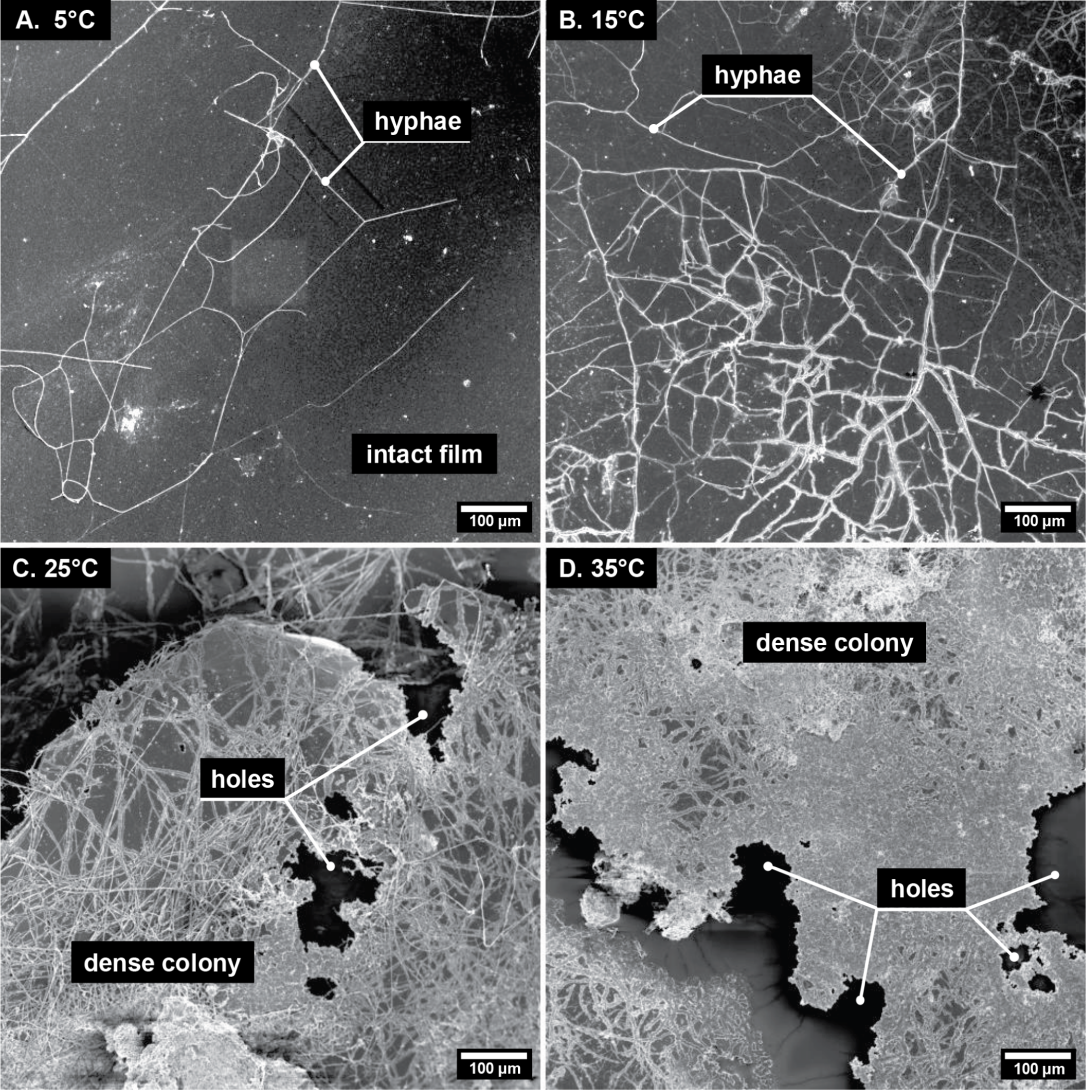
Selected scanning electron microscopy (SEM, secondary electron image, InLens detector) images of poly­(3-hydroxybutyrate-*co*-3-hydroxyhexanoate) films with a 3-hydroxyhexanoate content of 9% (PHBHH9) after incubation in soil LUFA 6S for 18 days at A) 5 °C, B) 15 °C, C) 25 °C, and D) 35 °C. Selected images for films incubated in LUFA 2.4 and 2.2 soils are provided in Figures S11–S12 in the Supporting Information. Exemplary hyphal structures, dense hyphal colonies, and holes in the films are labeled.

In principle, these hyphal structures may originate from both fungi, which have hyphae of diameters between 0.5 and 20 μm, and actinomycetota (i.e., filamentous bacteria with hyphae of diameters of 0.5 to 1 μm), and thus cannot be unequivocally delineated based on SEM images alone.^40,41^ Nonfilamentous-forming microorganisms were absent from the images. The imaging thus suggests that fungi and/or actinomycetota were more efficient in initial film colonization than nonfilamentous microorganisms. This finding likely reflects that hyphal structures can grow into air-filled spaces and onto “dry” film surfaces, whereas motile (nonfilamentous) bacteria and archaea would have required continuous water films on the PHBHH9 film surfaces for spatial movement.^42^

An increase in surface colonization and film disintegration with increasing temperature was also found for films incubated in LUFA 2.2 and 2.4 soils (Figures S8–S10). Yet, at a given tested temperature, film surface colonization and disintegration were most extensive in LUFA 6S and least in LUFA 2.2. For instance, while films incubated in LUFA 6S at 5 °C showed colonization (Figure 1A), films incubated at the same temperature in LUFA 2.2 showed few hyphae on only a small subarea of the total imaged surface (Figures S10 and S12). This qualitative image analysis thus suggests that the extent of PHBHH9 biodegradation decreased from LUFA 6S to 2.4 and finally 2.2.

#### Quantitative Image Analysis

Figure 2 shows the extent of colonization, expressed as the total length of hyphal structures per unit film surface area after 18 days of incubation in the three soils, as determined by SEM image analysis algorithms. The film area was categorized as holes when fully eroded and as colony when the region was so densely colonized that individual hyphae could not be distinguished by the hyphae identification algorithm, and the remaining area was designated as intact film. The extents of film surface colonization generally increased with increasing temperature from 5 to 35 °C with few exceptions (i.e., comparable hyphal lengths at 15 and 25 °C in LUFA 6S and comparable lengths at 25 and 35 °C for soils LUFA 2.4 and 2.2). While incubation at 5 °C for 18 days did not result in the formation of dense colonies, the film surface areas covered by dense colonies increased with increasing temperature from 15 to 35 °C for all three soils (Figure 2) (mainly at the expense of the areal contribution of intact film surface area). Then, the area of holes increased with increasing temperature above 25 °C. For LUFA 6S at 25 and 35 °C and for LUFA 2.4 at 35 °C, holes made up a substantial part of the imaged area (>8%, Figure 2A and B).

**Figure 2:**
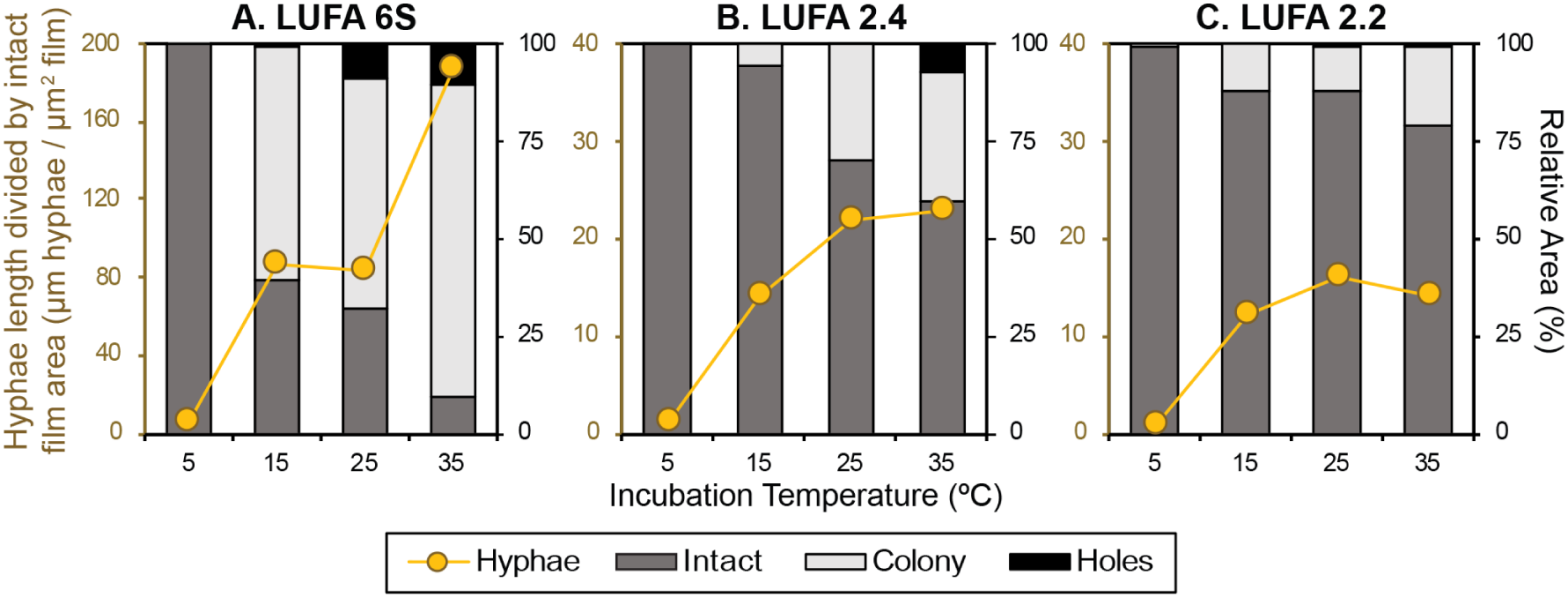
Quantitative analysis of hyphal colonization and disintegration of poly­(3-hydroxybutyrate-*co*-3-hydroxyhexanoate) films with a 3-hydroxyhexanoate content of 9% (i.e., PHBHH9) incubated in soils A) LUFA 6S, B) LUFA 2.4, and C) LUFA 2.2 at temperatures of 5, 15, 25, and 35 °C for 18 days, based on scanning electron microscopy (SEM) images of the film surface and image analysis algorithms.

Overall, this quantitative analysis of film colonization and disintegration confirms the above qualitative assessment: the extents of film colonization, surface erosion, and film disintegration increased with increasing incubation temperature and, at a given temperature, decreased from soil LUFA 6S to 2.4 and 2.2. Furthermore, the SEM image analysis revealed that hyphal growth, dense colonization, and film disintegration occurred simultaneously and nonuniformly across the film surfaces. This finding implies that polymer biodegradation in soils at any given time is highly spatially variable.

### PHBHHx Mass Loss Dynamics during Biodegradation

Figure 3 shows the residual mass of PHBHH9 powder incubated in LUFA 6S (3A), LUFA 2.4 (3B), and LUFA 2.2 (3C) soils over time at incubation temperatures ranging from 5 to 35 °C. Although we did not respirometrically monitor biodegradation to carbon dioxide (which was not possible for the number of samples), we consider the measured mass loss to accurately reflect biodegradation. Enzymatic or slow abiotic hydrolysis of PHBHHx is expected to yield low molecular weight oligomers and monomers, which are readily assimilated by microorganisms.^43–45^ We found no indication in the ^1^H NMR spectra that low molecular weight products accumulated in the soils (Figure S3). As expected, the rates of PHBHH9 mass loss, increased with increasing incubation temperature. At 35 °C, PHBHH9 biodegradation was fastest and almost complete within 28 (LUFA 6S), 42 (LUFA 2.4), and 49 days (LUFA 2.2) of incubation. In contrast, complete biodegradation at 5 °C required approximately one year in LUFA 6S but was not attained during the one-year incubations in LUFA 2.4 and 2.2 with 20 and 75% of the added PHBHH9 masses remaining, respectively.

**Figure 3:**
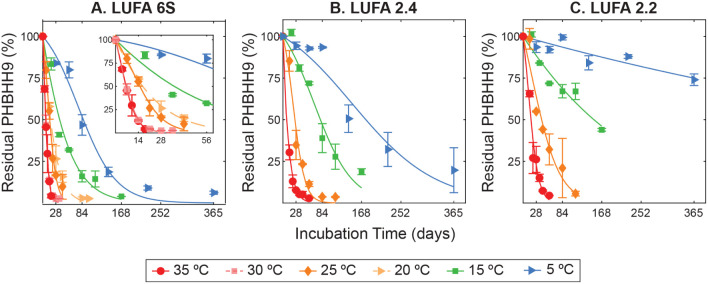
Residual mass of poly­(3-hydroxybutyrate-*co*-3-hydroxyhexanoate) with a 3-hydroxyhexanoate content of 9% (i.e., PHBHH9) versus incubation time in soils A) LUFA 6S, B) LUFA 2.4, and C) LUFA 2.2 at different temperatures (5, 15, 25, and 35 °C and, additionally, 20 and 30 °C in LUFA 6S) expressed in mass percent (%) of the initially added PHBHH9 mass. Symbols and error bars represent averages and standard deviations in residual PHBHH9 masses of triplicates, respectively. The solid lines represent fits of the shoulder-log linear kinetic model to the experimental data. Fitted model parameters are provided in Table 1.

For a quantitative analysis of biodegradation rates, we used a shoulder-log linear kinetic model to fit the residual mass data and obtain the duration of the initial lag phase (*L*) and the pseudo-first order biodegradation rate constant (*k*). The model fits generally described the data well: 33 out of 40 model data sets fitted yielded *R* ^2^ values > 0.90 with 29 fits having *R* ^2^ values > 0.95 (Table S3). We compared the quality of the fits of the shoulder-log linear model to the quality obtained by a simple pseudo-first order kinetic model (i.e., see eq 1, when *L* is set to 0 day). In general, including *L* as a fitting parameter significantly improved the data fits based on a comparison of Akaike information criterion (AIC), Bayesian information criterion (BIC), chi-squared, and −2log­(likelihood) values (Tables S3–S4). We note that biodegradation in LUFA 2.2 at 5 and 15 °C proceeded so slowly that the model fits of the lag phase had high uncertainties. This may be explained by the lag phase in this soil being greater than the length of the incubation (>1 year). Alternatively, the pseudo-first order biodegradation rate constant is so low, it is indistinguishable from the slow biodegradation during the lag phase. The lag phase was included (*L* > 0) in the model if it statistically significantly improved the model fit (its *p*-value was < 0.05). Fitted parameters are shown in Table 1 and model fits are shown in Figure 3 as solid or dashed lines.

**Table 1:** Model Parameters and Quality of Fit Criteria Obtained by Fitting the Shoulder-Log Linear Kinetic Model to the Residual Poly­(3-hydroxybutyrate-*co*-3-hydroxyhexanoate) (PHBHHx) Mass Data with a 3-Hydroxyhexanoate Content of 9 mol% (PHBHH9) in Soils LUFA 6S, 2.4, and 2.2^a^

		Pseudo-first order rate constant (*k*, d^–1^)		Lag phase (d)		
Soil	Temperature (°C)	Value	*p*-value	Value	*p*-value	*R* ^2^
**LUFA 6S**	5	0.025 ± 0.007	0.020	82 ± 8	0.001	0.98
	15	0.020 ± 0.002	0.0002	0^b^	0.58	0.97
	20	0.043 ± 0.002	<0.00001	0^b^	0.16	1.00
	25	0.11 ± 0.02	0.004	13 ± 1	0.001	0.99
	30	0.20 ± 0.02	0.001	5 ± 1	0.002	1.00
	35	0.22 ± 0.01	0.00004	5 ± 0	0.00004	1.00
**LUFA 2.4**	5	0.011 ± 0.005	0.069	164 ± 128	0.004	0.93
	15	0.025 ± 0.008	0.034	74 ± 10	0.002	0.96
	25	0.081 ± 0.024	0.026	24 ± 4	0.002	0.96
	35	0.088 ± 0.003	<0.00001	0^b^	1	1.00
**LUFA 2.2**	5	0.0008 ± 0.0001	0.002	0^b^	1	0.78
	15	0.0045 ± 0.0004	0.0002	0^b^	0.84	0.93
	25	0.034 ± 0.015	0.080	31 ± 13	0.076	0.95
	35	0.096 ± 0.029	0.028	14 ± 4	0.016	0.97

a The uncertainties of fitted parameters are provided as ± the standard error of each fitted parameter. Full statistical analysis and model fits for all experiments performed are provided in Tables S3–S4.

b Denotes that the *p*-value for *L* was > 0.05, thus indicating that *L* did not statistically significantly improve the model fit and therefore *L* was set to 0.

#### Effect of Incubation Temperature on the Initial Lag Phase

The duration of the biodegradation lag phase in LUFA 6S and 2.4 tended to decrease with increasing temperature (Figure 3; Table 1). For instance, in LUFA 6S, the lag phase decreased from close to 90 days at 5 °C to only 5 days at 35 °C. Among the tested soils, the lag phases were shorter for LUFA 6S across the tested temperature range (with few exceptions due to temporal resolution in the initial stages of the kinetic data) and longer for LUFA 2.4 (Table 1; Table S3). For instance, the lag phase in LUFA 2.4 at 5 °C was 164 days and thus about twice as long as for LUFA 6S. In contrast, the impact of temperature on the biodegradation lag phase in LUFA 2.2 was less clear: the relatively small decreases in the residual PHBHH9 mass with incubation time impeded accurate fitting of the lag phase at 5 and 15 °C. Still, a decrease in the lag phase in LUFA 2.2 was observed from 25 to 35 °C, with data sets being well fitted by the model. As expected, these lag phase values are larger than those at the same temperatures in LUFA 6S and LUFA 2.4.

The durations of fitted lag phases were in overall good agreement with the results from film imaging after 18 days of soil incubation. At 5 °C in LUFA 6S, and at 5 and 15 °C in LUFA 2.4, fitted lag phase values exceeded 18 days, consistent with SEM images showing only a few hyphae on the film surfaces and only sporadic colonies (Figures 1 and 2). Starting at 15 °C for LUFA 6S and at 25 °C for LUFA 2.4 and 2.2, lag phases were comparable to or shorter than the 18 days of film incubation, in agreement with extensive film colonization and disintegration observed under these conditions (Figures 1 and 2). This agreement supports that microbial colonization dynamics of the films after their addition to the soils largely contributed to the initial lag phase observed in the PHBHHx powder mass loss.

#### Effect of Incubation Temperature on Biodegradation Rates

Increasing temperatures from 5 to 35 °C resulted in continuous and pronounced increases in the pseudo-first order PHBHHx biodegradation rate constants (k) for all three soils (Table 1). The biodegradation rate constants across soils increased by a factor of 8 to 10 from 5 to 35 °C. The largest relative increase in k per 10 °C increase occurred from 15 to 25 °C, implying that soil microbial activity was most sensitive to temperature over this range for the three studied temperate soils. Largely consistent with the differences in the lag phases for the three soils, rate constants were highest in LUFA 6S, followed by LUFA 2.4, and finally LUFA 2.2.

The increase in PHBHHx biodegradation rate constants in the three studied soils with increasing temperature is consistent with previous reports of positive temperature effects on the biodegradation of polyhydroxybutyrate, polyhydroxybutyrate-*co*-hydroxyvalerate, and of a commercial biodegradable feedstock pellets containing polybutylene adipate-*co*-terephthalate (PBAT) in soil.^46–48^ Increasing PHBHHx biodegradation rate constants with increasing temperature can be rationalized by its increasing microbial metabolic activity and replication^9,19,49^ as well as the activity of extracellular microbial esterases involved in the breakdown of PHBHHx.^13,14^ It is likely, however, that had temperatures been increased further, biodegradation rates would have started to decrease above a critical temperature due to thermal inactivation of extracellular enzymes and by moving out of the temperature range of optimal growth of soil microorganisms. The highest tested temperature of 35 °C may have approached the critical temperature given the smaller relative increases in biodegradation rates in LUFA 6S and LUFA 2.4 between 25 and 35 °C as compared to between 15 and 25 °C.

The differences in the duration of initial lag phases and subsequent PHBHHx biodegradation rate constants among the three soils suggested that the abundance and activity of PHA degraders and their extracellular esterases (i.e., PHAses) involved in PHBHHx breakdown decreased from LUFA 6S to LUFA 2.4 and LUFA 2.2. This observation is consistent with previous studies that observed soil-specific differences in PHA biodegradation.^32,50,51^ The tested soils are expected to have different microbial community compositions, given their different soil types and physicochemical properties (Table S2). One possible explanation is that the slightly acidic pH of 5.6 in LUFA 2.2 as compared to pH 7.3 and 7.5 for LUFA 6S and 2.4, respectively, contributed to the low biodegradation of PHBHHx in LUFA 2.2 (Figures S13–S14). This explanation is supported by the general observation that soil respiration (one key metric of soil activity) decreases with decreasing pH due to the lower abundance of bacterial species.^52,53^ Nonetheless, establishing that soil acidity (and associated shifts in microbial communities) impairs PHBHHx biodegradation would require testing of PHBHHx biodegradation in a larger set of soils spanning the tested pH range. Our data set shows that PHBHHx biodegradation is dependent on both soils and temperature.

#### Effect of 3-Hydroxyhexanoate Content of PHBHHx on Its Soil Biodegradability

The three tested PHBHHx variants (i.e., PHBHH5, PHBHH9, and PHBHH12 with 3-HH contents of 5, 9, and 12 mol %, respectively) showed comparable biodegradation dynamics across the tested temperature range for each of the three soils. For example, PHBHH5, PHBHH9, and PHBHH12 showed comparable biodegradation rates and extents in soil LUFA 6S at 15 °C (Figure 4A; Figure S15). Comparable soil biodegradability of the three PHBHHx variants in the tested soils is also consistent with the finding that 3-HH contents in the extracted residual PHBHHx remain constant over the course of biodegradation (Figure 4B). We note that in a small subset of PHBHH12 samples, there is overlap with the 3-HH content of PHBHH9. There is greater uncertainty in the measured 3-HH mol % values for the PHBHH12 samples due to greater overlap between the 3-HH and 3-HB peaks, resulting in limitations in the deconvolution of the these overlapping ^1^H NMR peaks. Nonetheless, if the biodegradation of PHBHHx had depended on the 3-HH content, then the latter likely would have changed in an analogous manner with an increasing extent of biodegradation.

**Figure 4:**
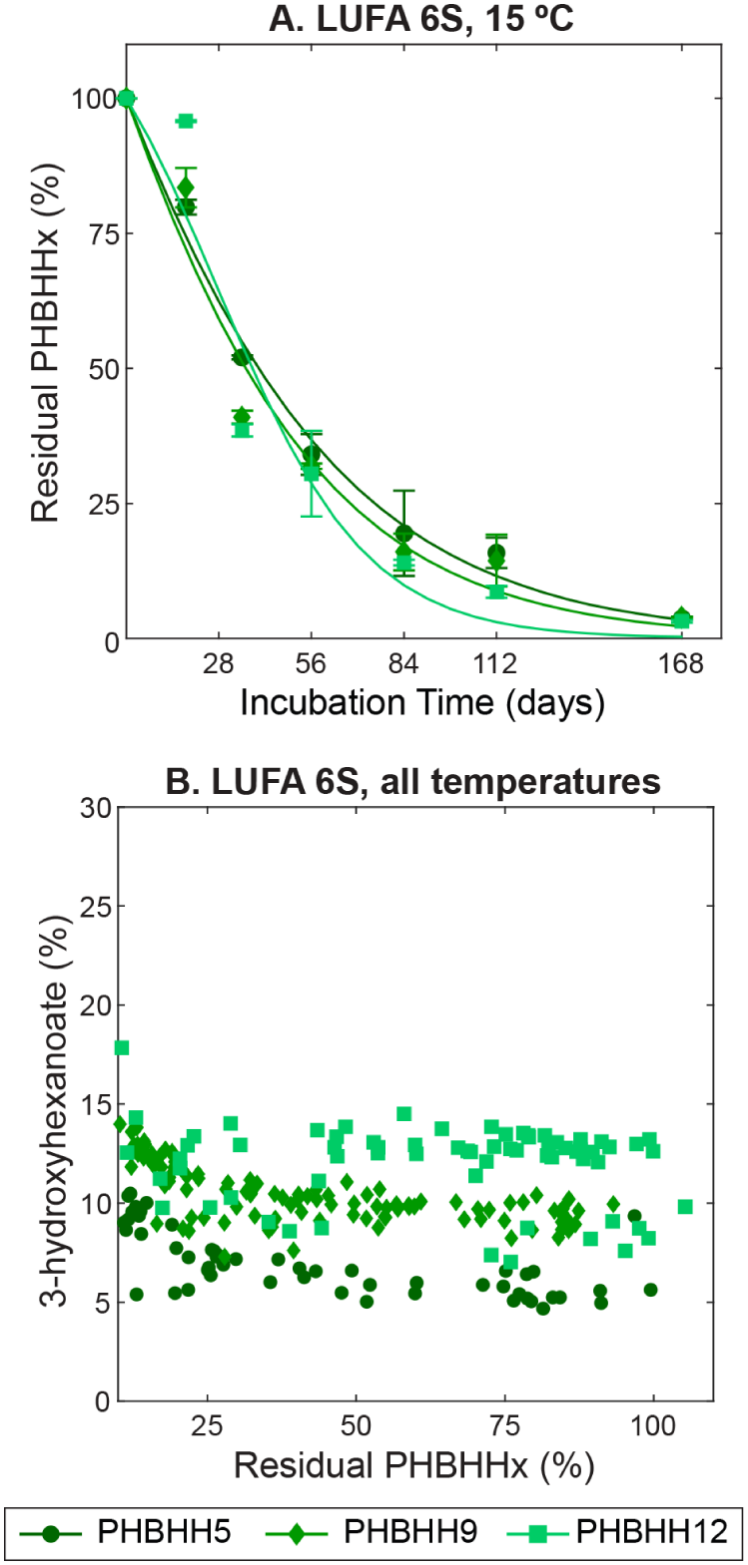
Comparison of the biodegradation dynamics of the three tested poly­(3-hydroxybutyrate-*co*-3-hydroxyhexanoate) variants with increasing 3-hydroxyhexanoate (3-HH) contents of 5 (PHBHH5), 9 (PHBHH9), and 12% (PHBHH12). (A) The biodegradation dynamics of the three PHBHH variants in LUFA 6S soil at 15 °C. (B) Quantified 3-hydroxyhexanoate contents (expressed in mole percent (%) of total 3-hydroxybutyrate and 3-hydroxyhexanoate) in the extracted, residual PHBHHx over the course of biodegradation of PHBHHx (expressed in mass percent (%) of the initially added PHBHHx mass) in LUFA 6S at all temperatures tested. Uncertainties of the measured 3-hydroxyhexanoate (3-HH) contents are generally on the order of 1% when the concentration of PHBHHx is high but increase with decreasing PHBHHx concentration (i.e., <0.67 mg mL^–1^ in the measured extract) due to challenges in spectral deconvolution.

Prior studies reported increasing PHBHHx enzymatic hydrolyzability and increasing PHBHHx biodegradability in soil, sludge, and compost with increasing 3-HH (= x) contents relative to pure poly­(3-hydroxybutyrate) (PHB).^27,30,50,54^ This finding was ascribed to decreasing PHBHHx crystallinity with increasing 3-HH contents, particularly when comparing PHBHHx to pure PHB.^27^ The crystallinity of pure PHB is estimated at 60%; we measured lower crystallinities for the PHBHHx used herein, ranging from 52% (PHBHH5) to 29% (PHBHH12) (Table S1).^55^ Although increasing enzymatic hydrolyzability and hence biodegradation rates with decreasing crystallinity have been reported for other polyesters, our results suggest that potential decreases in the crystallinity from PHBHH5 to PHBHH12 were insufficient to lead to measurable differences in overall biodegradation rates.

#### Analysis of Temperature-Dependent Rate Constants Using Arrhenius Rate Law

Figure 5 shows the natural logarithm of the fitted biodegradation rate constants of PHBHH9, ln­(*k*), plotted versus inverse absolute temperature and the respective fits by the Arrhenius rate law (solid lines; eq 2):

$$\ln\left(k\right)=-\left(\frac{E_{a}}{R}\right)\cdot \frac{1}{T}+\ln\left(A\right)$$ 2

where *E*_a_ (J mol^–1^) is the apparent activation energy for the PHBHHx biodegradation, *R* (= 8.314 J (K mol)^−1^) is the universal gas constant, *T* (K) is the absolute temperature, and *A* is the pre-exponential factor. The Arrhenius rate law generally described the experimental data well, especially considering that this law is intended to describe the temperature dependence of simple, one-step reactions.^56,57^ Observed deviations were most likely related to the multistep nature of biodegradation: microbial colonization, extracellular enzymatic PHBHHx hydrolysis, and cellular uptake and metabolic utilization of the PHBHHx hydrolysis products. Specifically, nonlinearity is best exemplified in the LUFA 6S data, where there is clear curvature at both the high (i.e., 30 to 35 °C) and low (i.e., 5 to 15 °C) temperature changes, where biodegradation is less sensitive to temperature changes. Despite the fits not being perfect, they provided approximate activation energies *E*_a_, and, thereby, estimates for the temperature coefficients, *Q*_10_, of PHBHHx biodegradation in the tested soils. The *Q*_10_ value is commonly used in biogeochemical and environmental microbiology studies to describe the factor change in substrate turnover per temperature change of 10 °C.^58–60^

**Figure 5:**
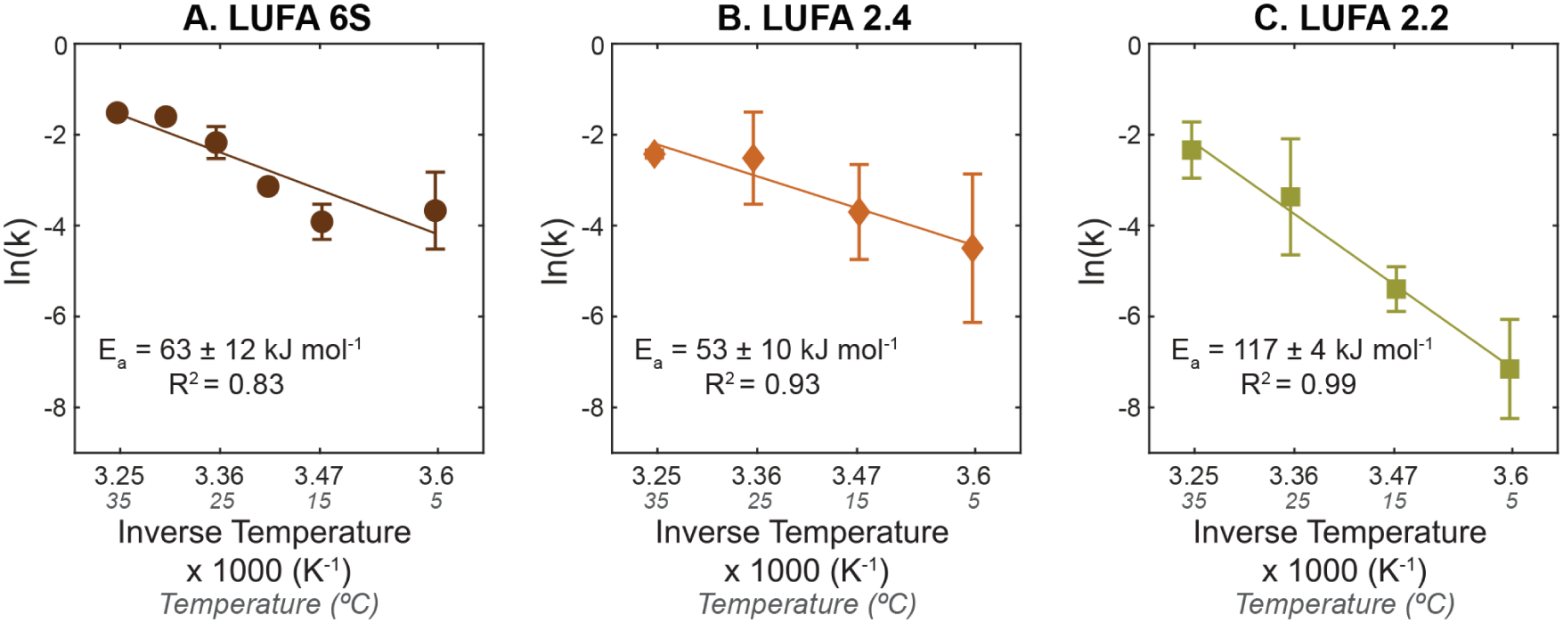
Plots of the natural logarithm of the fitted biodegradation first-order rate constants, ln­(*k*), of poly­(3-hydroxybutyrate-*co*-3-hydroxyhexanoate) with 9% 3-hydroxyhexanoate (i.e., PHBHH9) in soils LUFA 6S (A), LUFA 2.4 (B), and LUFA 2.2 (C) versus the inverse of the absolute temperature. The solid lines represent data fits by the Arrhenius rate law. The error bars represent the standard error of fitted rate constants *k* and likely underestimate the uncertainty of these values.

For LUFA 6S, the Arrhenius rate law adequately described the rate constant data (*R* ^2^ = 0.83), resulting in an *E*_a_ estimate of 63 ± 12 kJ mol^–1^ (Figure 5A). Constraining the fit of the Arrhenius rate law to the rate data between 15 and 30 °C increased the activation energy to *E*_a_ = 115 ± 7 kJ mol^–1^ and substantially increased the overall fit quality (i.e., *R* ^2^ = 0.99). Fits of the Arrhenius rate law to all biodegradation rate constant data for LUFA 2.4 resulted in reasonable fits with *E*_a_ = 53 ± 10 kJ mol^–1^ (*R* ^2^ = 0.93) (Figure 5B). Comparable to *k* values in LUFA 6S, the largest relative increase in *k* per increase in temperature of 10 °C occurred between 15 and 35 °C in LUFA 2.4, as compared to the temperature increases from 25 to 35 °C (Figure 5B). When fitting only the 5 and 25 °C rate constant data, the activation energy increased to *E*_a_ = 69 kJ mol^–1^. For LUFA 2.2, fitting all rate constant data yielded *E*_a_ = 117 ± 9 kJ mol^–1^ with an overall good fit (*R* ^2^ = 0.99). The activation energy for biodegradation of the two remaining PHBHH5 and PHBHH12 variants showed similar activation energies for a given soil (within uncertainty; Table S5; Figure S16). However, fitted *E*_a_ values for the PHBHHx variants differed between the soils.

The fitted *E*_a_ corresponds to *Q*_10_ values (average factor change of 0 to 40 °C) of 2.2 (for *E*_a_ = 53 kJ mol^–1^ in LUFA 2.4) to 5.1 (for *E*_a_ = 117 kJ mol^–1^ in LUFA 2.2).^61^ A decrease in incubation temperature from 25 to 15 °C thus is expected to lower biodegradation rates in soils approximately 2- to 5-fold (in addition to increasing the length of the lag phase). The calculated *Q*_10_ values support that PHBHHx biodegradation in soils was highly temperature dependent and, therefore, that PHBHHx biodegradation tests conducted in laboratory incubations at 25 °C are expected to overestimate PHBHHx biodegradation rates in temperate soils under lower temperature *in situ* conditions in the field.

The range of *E*_a_ = 53–117 kJ mol^–1^ for PHBHHx biodegradation in the three soils between 15 - 25 to 30 - 35 °C is in good agreement with previously reported activation energies for polyester hydrolysis and biodegradation. Pischedda et al. (2019) estimated an *E*_a_ = 108.7 kJ mol^–1^ for the mineralization of a PBAT-dominated mulch pellet in a sandy loam agricultural soil over a temperature range of 15 to 28 °C (although CO_2_ formation originating from the PBAT and the other pellet components, such as starch, could not be delineated).^47^ The finding of differing *E*_a_ values for PHBHHx biodegradation between the three tested soils implies that not only overall PHBHHx biodegradation dynamics but also its temperature dependence are soil specific. This specificity likely reflects differences in the microbial communities: types and activities of PHBHHx-hydrolyzing enzymes and biodegrading microorganisms.

## Environmental Implications

This work demonstrates that temperature is a critical environmental factor controlling the rate of PHBHHx biodegradation in soils: as temperature decreases, the length of the initial lag phase prior to the onset of PHBHHx biodegradation increases, and the subsequent biodegradation rate constants decrease. Evidence is provided that the initial lag phase is linked to the time needed for the microbial colonization of the PHBHHx surface. The finding by SEM image analysis that fungi and/or filamentous bacteria dominated the biomass pool on the PHBHHx surface during initial colonization points to limitations in colonization by motile bacteria, possibly due to the absence of continuous water films. Increasing PHBHHx biodegradation rates with increasing temperature are expected to primarily reflect increasing enzymatic and microbial activity. Activation energies obtained from applying the Arrhenius rate law suggest temperature coefficients Q_10_ for PHBHHx biodegradation of 2 to 5, suggesting substantially slower biodegradation of PHBHHx in soils at environmental temperatures (i.e., 2- to 5-fold slower at 15 °C and 4- to >20-fold slower at 5 °C). These values need to be considered estimates that, as shown herein, also vary between soils (even when collected from the same climatic region). Future work is needed to establish causation for differences in activation energies and thus the temperature dependence of polyester biodegradation between soils, which may require quantifying activities of key extracellular enzymes and microbial degraders. These tests may also include soils from different climatic zones to assess if and how the natural temperature ranges of the soil affect the temperature dependence of polyester biodegradation.

Given that the tested PHBHHx are polyesters, strong temperature dependencies are also expected for the biodegradation of other commercially important biodegradable polyesters, including those used in commercial products, such as biodegradable mulch films. However, the extension of such temperature dependencies to other polyesters must still be experimentally verified. These dependencies are needed to extend capabilities to predict biodegradation rates in the field under *in situ* conditions based on rates determined in laboratory incubations at higher temperatures. Ultimately, parallel laboratory incubations at different but constant temperatures (to establish the temperature dependence) and field incubations in the same soils coupled to close monitoring of soil temperature variations will allow for assessing the extent to which temperature controls biodegradation rates in the field. The systematic assessment of temperature on PHBHHx biodegradation in soils provided herein is a first step toward advancing a detailed understanding of the dependence of polyester biodegradation on critical abiotic system factors, including soil water content and pH. This understanding is critical to obtain predictive power for biodegradation rates under *in situ* field conditions and to select polyesters for biodegradable products with biodegradability characteristics that ensure performance both during use and during biodegradation in soils after use.

## Supplementary Material



## Notes

PHBHHx characterization, image analysis details, SEM images, and additional kinetic plots and tables (PDF)
