# Supplementary material for: Temperature Dependence of Poly(3-hydroxybutyrate-co-3-hydroxyhexanoate) Biodegradation in Agricultural Soils
Source: Environ Sci Technol. 2026 Feb 23;60(9):7183–93. doi: 10.1021/acs.est.5c08707 (PMC12980838; doi:10.1021/acs.est.5c08707)
Supplement: Supplementary file 1 [file es5c08707_si_001.pdf]

Temperature dependence of poly(3-hydroxybutyrate-co-3-hydroxyhexanoate) biodegradation in agricultural soils

Supporting Information

Juliana R. Laszakovits[a], Silvan Arn[a], Ralf Kägi[b], Silvan Liechti[a], Flora Wille[a], Kristopher McNeill[a,*], Michael Sander[a,b,*]

[a]Institute of Biogeochemistry and Pollutant Dynamics (IBP), Department of Environmental Systems Science, ETH Zuerich, 8092 Zuerich, Switzerland
[b]Eawag, Swiss Federal Institute of Aquatic Science and Technology, Ueberlandstrasse 133, CH-8600 Duebendorf, Switzerland
*Corresponding authors:
Kristopher McNeill; email: kristopher.mcneill@env.ethz.ch
Michael Sander; email: michael.sander@env.ethz.ch

**Contents**	**Page**
Text S1: Scanning electron microscopy analysis	S1
Text S2: Quantitative ^{1}H nuclear magnetic resonance analysis	S3
Table S1: PHBHHx key physicochemical properties	S5
Table S2: Soil properties	S5
Table S3: Summary of parameters fitted by kinetic shoulder-log linear model.	S6
Table S4: Summary of parameters fitted by kinetic shoulder-log linear model for L = 0 days	S8
Table S5: Summary of Arrhenius fit parameters for different PHBHHx variants	S10
Figure S1: Example PHBHHx ^{1}H NMR spectra	S11
Figure S2: Example PHBHHx ^{1}H NMR spectra with LUFA 6S background	S12
Figure S3: Example soil background in ^{1}H NMR spectra	S13
Figure S4: Differential scanning calorimetry (DSC) scans	S14
Figure S5: PHBHHx particle size distribution	S15
Figure S6: Illustration of the image analysis for hyphal length	S16
Figure S7: Spike recovery PHBHHx quality control data	S18
Figure S8: Mosaic image of PHBHH9 films incubated in LUFA 6S soil	S20
Figure S9: Mosaic image of PHBHH9 films incubated in LUFA 2.4 soil	S21
Figure S10: Mosaic image of PHBHH9 films incubated in LUFA 2.2 soil	S22
Figure S11: Examples of individual images of PHBHH9 films incubated in LUFA 2.4 soil	S23
Figure S12: Examples of individual images of PHBHH9 films incubated in LUFA 2.2 soil	S23
Figure S13: Effect of soil on PHBH biodegradation	S24
Figure S14: Comparison of biodegradation in LUFA 2.2 and LUFA 2.4 soils relative to in LUFA 6S	S25
Figure S15: Effect of 3-hydroxyhexanoate percentage on PHBHHx biodegradation	S26
Figure S16: Arrhenius activation energies for three PHBHHx variants	S27
Text S3: Image analysis details	S28
References	S35

Pages: 35, 16 figures, 5 tables

Text S1: Scanning electron microscopy analysis

Data acquisition: Rectangular areas with dimensions ranging from 5.8 x 5.8 mm to 8.2 x 8.2 mm were imaged by an automated collection of individual SE images in a regular arrangement using the ATLAS software package. The resolution of the individual images was 100 nm pixel^{-1} and the image size was set to 8192 pixels. The dwell time per pixel was 0.2 µs and every line was averaged over 5 consecutive scans. Individual images obtained from both SE detectors (SE2 referring to the images obtained from the Everhart–Thornley detector) were recorded simultaneously and were stored for subsequent processing. For every individual image, an autofocus routine was applied to account for the variable topography of the mounted PHBHH9 films.

Quantification of total length of individual hyphae on films. All SE images collected for a given film were downsized to 512 x 510 pixels (pixel size = 1.6 µm x 1.6 µm) and subsequently stitched into one mosaic image using Fiji's "Grid/Collection stitching" function (version ImageJ 2.14.0/1.54f). A rectangular mask was then placed over this film image to define the subarea that was used for image analysis below (approximate size = 25,606 µm^2).

We first determined the total length of hyphae on the film surface that were detectable by the following procedure (we note that this does not include hyphae in dense colonies; see below). First, using the InLens SE images, we enhanced the image contrast through equalization and normalization of the pixel intensity histogram. Second, we used the '*remove outliers function'* (Text S3) to delineate film background (dark colored in images) from hyphae and other bright features (i.e., 'outliers') on the film. This treatment was successful only for 'isolated' hyphal features on the otherwise bare film surface but not for densely colonized areas. We then subtracted the background from the analyzed image, resulting in film background appearing black and all outliers as brighter pixels. Third, we again enhanced the contrast again (see above). Forth, we cut the obtained image in sub-images that were processable by the ridge detection plugin.[1] A binary image showing the detected ridges was subsequently filtered to remove ridge features with areas <40 µm^2. Fifth, the ridge detection plugin was again applied to generate an image overlay (a feature in ImageJ).[2] Sixth, the hyphae length on each sub-image was computed from the respective image overlay and subsequently summed over all sub-images to compute the total hyphae length. Seventh, the image overlays and binary images of all sub-images were stitched to re-obtain the full analyzed image. The resulting images were projected onto the original image to visually confirm that hyphae were correctly captured by this procedure. Illustration of this procedure can be found in Figure S5. The total length of detectable hyphae (μm_{hyphae}) on each film surface was normalized to the total analyzed film area minus the area of film holes (µm^2) (see below).

Quantification of total area of dense colonies on films. Dense microbial colonies on the films were detected using a python script involving Canny edge detection (Text S3) to obtain a binary image of detected contrast edges (i.e., lines). Lines close to one another (distance <16 µm) were 'fused' into single features using morphological closing (Text S3). This operation resulted in conversion not only of densely colonized areas into single large features but also of isolated hyphae (with two edges) into single 'thin' lines. To remove the latter from actual dense colonies, a morphological opening with a circular kernel (radius 32 µm) was applied. Finally, only objects with areas above a threshold of 25'606 um^2 (corresponding to approximately 10,000 pixels) were

considered dense colonies. The areas of individual colonies on a film were summed to obtain the total area of colonies. This area (um^2 colonies) was normalized to the total analyzed area of the image (um^2).

Quantification of total area of holes in films. Holes were visually identified on SE2 images after contrast enhancement and subsequently marked manually with the Labkit plugin in ImageJ.[3] The macros for this procedure are given in Text S3. The total area of holes (um^2 holes) was normalized to the total analyzed area of the image (um^2).

Text S2: Quantitative ^{1}H nuclear magnetic resonance analysis

The dried extracts were reconstituted in 3 mL deuterated chloroform that contained ~1 mg mL^{-1} dimethoxybenzene (DMB). ^{1}H NMR spectra were collected as described in the main text. The spectra were processed in Mestrenova.[4] First, the spectra were referenced to the solvent peak (residual non-deuterated chloroform at chemical shift δ= 7.26 ppm). The phase was corrected automatically and, if required, further manually corrected. The baseline was corrected by multipoint baseline correction. Finally, the relevant peaks were integrated: DMB aryl proton peaks were integrated between δ = 6.75 and 6.90 ppm, the area of one 3-hydroxybutyrate monomeric unit proton between δ = 2.55 and 2.68 ppm, and the area of one 3-hydroxybutyrate monomeric unit proton and two 3-hydroxyhexanoate monomeric unit protons between δ = 2.40 and 2.55 ppm. The relevant regions are visualized in Figure S1.

To quantify residual PHBHHx, a conversion factor was determined for every spectrum to relate the area to the moles of protons present in the sample according to Equation S1:

$$f = \frac{\frac{V \times C_{DMB} \times H_B}{MW_{DMB}}}{A_{H_B}} \qquad \text{Equation S1}$$

where f is the conversion factor between mmol of H and signal intensity (area) in the NMR spectrum, V is the volume of $CDCl_3$ added to the sample (mL), C_{DMB} is the concentration of DMB (mg mL^{-1}), MW_{DMB} is the molecular weight of DMB (138 g mol^{-1}), H_B (= 4) is the number of aryl protons per DMB molecule, and A_{HB} is the area from the DMB aryl Hs (δ = 6.75 to 6.90 ppm).

The area of one proton of the 3-hydroxybutyrate (A_{HB}) monomeric unit was set to be the area between δ = 2.55 and 2.68 ppm according to Equation S2:

$$A_{HB}=A_{2.55\text{-}2.68ppm} \qquad \text{Equation S2}$$

The area of two 3-hydroxyhexanoate monomeric unit protons (A_{HH}) was subsequently determined using A_{HB} according to Equation S3:

$$A_{HH}=A_{2.55\text{-}2.4ppm}-A_{HB} \qquad \text{Equation S3}$$

Where $A_{2.55\text{-}2.4ppm}$ is the area between δ = 2.40 and 2.55 ppm.

The total mass of the 3-hydroxybutyrate monomeric units (m_{HB}, mg) was used with the conversion factor (f) determined by Equation S1 and the molecular weight of the 3-hydroxybutyrate monomeric unit (MW_{HB}, 86 g mol^{-1}; weight of 3-hydroxybutyrate minus OH) as follows:

$$m_{HB}=A_{HB} \times f \times MW_{HB} \qquad \text{Equation S4}$$

The total mass of the 3-hydroxyhexanoate monomeric units (m_{HH}, mg) was used with the conversion factor (f) determined by Equation S1 and the molecular weight of the 3-hydroxybutyrate monomeric unit (MW_{HB}, 114 g mol^{-1}; weight of 3-hydroxybutyrate minus OH) using:

$$m_{HH}=\frac{A_{HH}}{2} \times f \times MW_{HH} \qquad \text{Equation S5}$$

Note that the 2 accounts for the two protons present that give rise to the area calculated in Equation S3.

Finally, the masses of the two subunits were used to calculate the total mass of PHBHHx (m_{PHBHHx}, mg) extracted from the soil,

$$m_{PHBHHx}=m_{HB}+m_{HH} \qquad \text{Equation S6}$$

Finally, two key measures could be calculated: the residual PHBHHx (%) in the sample (Equation S7) and the molar percentage of 3-hydroxyhexanoate (3-HH) in the sample (Equation S8):

$$\text{Residual }(\%)=\frac{m_{PHBHHx}}{m_{initial.PHBHHx}}\times 100\% \qquad \text{Equation S7}$$

Where $m_{initial,PHBHHx}$ is the mass of PHBHHx added to the soil (mg).

$$\text{3-HH }(\%)=\frac{m_{HH}/MW_{HH}}{m_{HH}/MW_{HH}+m_{HB}/MW_{HB}}\times 100\% \qquad \text{Equation S8}$$

Table S1: PHBHHx key physicochemical properties

Representative ^{1}H NMR spectra can be found in Figure S1. Differential scanning calorimetry (DSC) scans are provided in Figure S4. Particle size distribution of the three powders used is presented in Figure S5.

Abbreviation	PHBHH5	PHBHH9	PHBHH12
**3-hydroxyhexanoate (mol %)**	5	9	12
**Molecular weight (kDa)^**	620	590	650
**Polydispersity^**	2.3	2.3	2.3
**Literature Crystallinity (%)***	41.6 ± 5	30.1 ± 5	30.7 ± 5
**Measured Crystallinity (%)#**	52	42	29
**Median particle size (µm)§**	123	106	268

^Gel permeation chromatography with refractive index detection was used to determine weight-average molecular weight and polydispersity for PHBHHx dissolved in chloroform against polystyrene standards.

*Crystallinity from literature values.[5] Alata *et al.* measured crystallinity as a function of aging so we consider their reported crystallinities determined on day 1 of their experiments as equivalent to the initial crystallinity of our PHBHHx samples. Alata *et al.* did not report a 9% 3-HH sample, so we approximated this value based on their tested 10% 3-HH variant.

#Crystallinity determined by DSC for our PHBHHx samples. A Q1000 DSC module (TA instruments) was used to measure the first melt: - 30 to 200 °C at a heating rate of 10 °C min^{-1}.

§Particle size analysis was conducted by laser diffraction particles size analysis (Beckman Coulter, LS 13 320) using a universal liquid module to introduce the PHBHHx particles in a suspension of 4 g L^{-1}.

Table S2: Soil Properties

Soil properties were measured and provided by LUFA Speyer.

Property	LUFA 2.2	LUFA 2.4	LUFA 6S
**Soil Type**	Sandy loam	Loam	Clay
**Maximum water holding capacity (g/100g)**	43.4 ± 5.1	45.6 ± 2.7	41.4 ± 1.5
**Organic Carbon (%)**	1.77 ± 0.05	1.83 ± 0.25	1.55 ± 0.14
**Nitrogen (%)**	0.2 ± 0.06	0.23 ± 0.02	0.18 ± 0.01
**pH value (0.01 M $CaCl_2$)**	5.6 ± 0.3	7.5 ± 0.1	7.3 ± 0.1
**Cation exchange capacity (meq/100g)**	8.5 ± 2.0	17.6 ± 1.0	18.7 ± 1.2

Table S3: Summary of parameters fitted by kinetic shoulder-log linear model

The results are given for all experiments performed (three soils: LUFA 6S, LUFA 2.4, and LUFA 2.2); four temperatures (5, 15, 25, and 35 °C and two additional temperatures, 20 and 30 °C for LUFA 6S), and three PHBHHx variants (3-hydroxyhexanoate (3-HH) contents x = 5, 9, and 12%). The two model output parameters are: (1) pseudo-first order biodegradation rate constant, k (d^{-1}), given with t-stat value (for comparison with L = 0 d model), p-value (which gives significance of value to improving model fit) and (2) duration of the lag phase, L (days), also given also with t-stat and p-values. The following statistical parameters are given for every model fit: (1) R^2, which assesses the quality of the model fit but does not consider the number of fitting parameters; (2) Akaike information criterion (AIC), which gives a measure of model fit considering the number of fitting parameters but favors more complex models, (3) Bayesian information criterion (BIC), which gives a measure of model fit considering number of fitting parameters but favors simpler models, (4) Chi-squared (χ^2), which assesses the quality of the model fit, and (5) -2 log(likelihood), which measures how well the model fits the data. Overall, for most experiments, including the lag phase L resulted in a statistically significantly better fit based on comparison of R^2, AIC, BIC, and χ^2. For simplicity, we chose to set L = 0 d, when the p-value was > 0.05 for the lag phase parameter.

Soil	Temperature (ºC)	3-HH (%)	Pseudo-first order rate constant, k (d^{-1})			Lag phase, L (d)			R^2	AIC	BIC	χ^2	-2log (likeli-hood)
			Value	t-stat	p-value	Value	t-stat	p-value					
LUFA 6S	5	5	0.028 ± 0.009	3.03	0.039	125 ± 11	10.61	0.0004	0.96	29	29	1432	48
		9	0.025 ± 0.007	3.74	0.020	82 ± 8	9.93	0.001	0.98	25	24	516	43
		12	0.021 ± 0.004	4.92	0.008	102 ± 7	13.74	0.0002	0.99	22	22	72	40
	15	5	0.022 ± 0.005	4.62	0.010	16 ± 14*	1.15	0.32	0.99	18	18	1	33
		9	0.025 ± 0.011	2.26	0.086	15 ± 25*	0.60	0.58	0.97	27	26	9	45
		12	0.045 ± 0.023	1.94	0.12	34 ± 11	3.02	0.039	0.94	32	31	48	51
	20	9	0.054 ± 0.008	6.75	0.003	7 ± 4*	1.72	0.16	1.00	10	10	6	23
	25	5	0.209 ± 0.029	7.33	0.002	20 ± 1	22.56	0.00002	0.99	18	18	16 x 10^6	35
		9	0.11 ± 0.02	6.16	0.004	13 ± 1	9.46	0.001	0.99	18	18	11	31
		12	0.060 ± 0.019	3.16	0.034	0 ± 9*	0	1	0.99	18	18	68	35
	30	9	0.20 ± 0.02	9.96	0.001	5 ± 1	7.50	0.002	1.00	7	6	3	12
	35	5	0.15 ± 0.03	4.47	0.010	5 ± 2	2.83	0.050	0.99	19	19	2	32
		9	0.22 ± 0.01	19.72	0.00004	5 ± 0	20.23	0.00004	1.00	3	3	1	16

		12	0.21 ± 0.01	14.57	0.0001	6 ± 0	19.44	0.00004	1.00	8	7	1	21
LUFA 2.4	5	5	0.004 ± 0.005	0.81	0.46	35 ± 412*	0.08	0.94	0.83	34	33	18	54
		9	0.011 ± 0.005	2.46	0.069	164 ± 128	5.89	0.004	0.93	30	30	15	49
		12	0.006 ± 0.004	1.35	0.25	127 ± 130*	0.97	0.39	0.88	32	32	12	51
	15	5	0.036 ± 0.007	5.36	0.006	72 ± 4	16.65	0.0001	0.98	23	22	34	41
		9	0.025 ± 0.008	3.17	0.034	74 ± 10	7.38	0.002	0.96	27	27	13	46
		12	0.030 ± 0.006	4.97	0.008	78 ± 5	14.86	0.0001	0.98	23	23	7	41
	25	5	0.14 ± 0.02	6.20	0.003	22 ± 1	18.98	0.00005	0.99	19	19	21951	36
		9	0.081 ± 0.024	3.44	0.026	24 ± 4	6.84	0.002	0.96	26	25	174	50
		12	0.16 ± 0.07	2.39	0.075	27 ± 2	11.44	0.0003	0.96	30	29	95996	49
	35	5	0.093 ± 0.018	5.01	0.007	0 ± 4*	0	1	1.00	10	10	22	26
		9	0.088 ± 0.019	4.62	0.010	0 ± 5*	0	1	1.00	12	11	10	28
		12	0.063 ± 0.009	7.26	0.002	2 ± 4*	0.43	0.69	1.00	10	10	2	26
LUFA 2.2	5	5	0.001 ± 0.005	0.17	0.88	0 ± 8780*	0	1	0.66	24	24	2	42
		9	0.001 ± 0.004	0.22	0.84	0 ± 6538*	0	1	0.78	22	21	1	40
		12	0.001 ± 0.004	0.26	0.81	0 ± 4412*	0	1	0.73	25	24	2	43
	15	5	0.006 ± 0.003	1.86	0.14	82 ± 81*	1.01	0.37	0.97	18	18	1	35
		9	0.005 ± 0.005	1.04	0.36	43 ± 197*	0.22	0.84	0.93	24	24	2	42
		12	0.006 ± 0.004	1.60	0.19	116 ± 73*	1.59	0.19	0.96	19	19	1	37
	25	5	0.019 ± 0.007	2.63	0.058	5 ± 29*	0.19	0.86	0.98	23	23	4	43
		9	0.034 ± 0.015	2.33	0.080	31 ± 13*	2.38	0.076	0.95	29	29	7	48
		12	0.021 ± 0.005	4.12	0.015	34 ± 13*	2.70	0.054	0.98	20	20	1	37
	35	5	0.10 ± 0.02	6.31	0.003	16 ± 2	9.11	0.001	0.99	18	18	12	35
		9	0.096 ± 0.029	3.36	0.028	14 ± 4	4.04	0.016	0.97	25	25	9	43
		12	0.097 ± 0.024	3.96	0.017	18 ± 3	7.02	0.002	0.97	25	24	8	43

*L was set to 0 d, because $p > 0.05$

Table S4. Summary of parameters fitted by kinetic shoulder-log linear model for L = 0 days

The results are given for all experiments performed: three soils (LUFA 6S, LUFA 2.4, and LUFA 2.2), four temperatures (5, 15, 25, and 35 °C and two additional temperatures, 20 and 30 °C for LUFA 6S), and three PHBHHx variants (with 3-hydroxyhexanoate (3-HH) contents of x = 5, 9, and 12%). The model output parameter is a pseudo-first order biodegradation rate constant, k (d^{-1}), given with t-stat value and p-value. The following statistical parameters are given for every model fit: (1) R^2, which assesses the quality of the model fit but does not consider the number of fitting parameters; (2) Akaike information criterion (AIC), which gives a measure of model fit considering the number of fitting parameters but favors more complex models, (3) Bayesian information criterion (BIC), which gives a measure of model fit considering number of fitting parameters but favors simpler models, (4) Chi-squared (χ^2), which assesses the quality of the model fit, and (5) -2 log(likelihood), which measures how well the model fits the data. Overall, for most experiments, including the lag phase L in the fitting resulted in a better data fits (Table S3). For incubations at higher temperatures, where the lag phase was short, little difference is observed between the pseudo-first order rate constants.

Soil	Temperature (ºC)	3-HH (%)	Pseudo-first order rate constant, k (d^{-1})			R^2	AIC	BIC	χ^2	-2log(likelihood)
			Value	t-stat	p-value					
LUFA 6S	5	5	0.006 ± 0.001	5.01	0.004	0.89	33	32	19	54
		9	0.009 ± 0.001	6.74	0.001	0.94	29	29	14	50
		12	0.007 ± 0.001	6.63	0.001	0.94	29	29	13	50
	15	5	0.018 ± 0.001	18.02	<0.00001	0.99	17	17	1	36
		9	0.020 ± 0.002	9.25	0.0002	0.97	25	25	6	46
		12	0.020 ± 0.004	5.41	0.003	0.92	32	31	14	53
	20	9	0.043 ± 0.002	26.72	<0.00001	1.00	10	10	2	25
	25	5	0.045 ± 0.010	4.52	0.006	0.91	32	32	33	54
		9	0.052 ± 0.005	10.06	0.0002	0.96	24	24	6	39
		12	0.060 ± 0.004	14.96	0.00002	0.99	16	16	68	35
	30	9	0.124 ± 0.007	16.90	0.00001	0.99	15	14	3	25
	35	5	0.098 ± 0.007	14.46	0.00003	0.98	20	20	4	35
		9	0.122 ± 0.009	13.75	0.00004	0.98	21	20	7	36
		12	0.105 ± 0.009	11.93	0.00007	0.97	23	22	6	38
	5	5	0.004 ± 0.001	4.99	0.004	0.83	32	32	17	54

LUFA 2.4		9	0.004 ± 0.001	5.28	0.003	0.88	31	31	13	53
		12	0.003 ± 0.001	5.59	0.003	0.88	30	30	11	52
	15	5	0.010 ± 0.002	5.94	0.002	0.90	31	31	17	53
		9	0.009 ± 0.001	6.45	0.001	0.91	30	30	11	52
		12	0.009 ± 0.001	6.10	0.002	0.90	31	31	15	52
	25	5	0.038 ± 0.007	5.63	0.002	0.93	30	30	19	52
		9	0.031 ± 0.005	6.65	0.001	0.93	29	29	15	53
		12	0.029 ± 0.007	4.19	0.009	0.87	35	35	28	57
	35	5	0.092 ± 0.003	28.09	0.00000	1.00	8	8	22	26
		9	0.088 ± 0.003	25.65	<0.00001	1.00	10	10	10	28
		12	0.060 ± 0.002	37.39	<0.00001	1.00	8	8	1	26
LUFA 2.2	5	5	0.0008 ± 0.0002	4.85	0.005	0.66	22	22	2	42
		9	0.0008 ± 0.0001	6.15	0.002	0.78	20	19	1	40
		12	0.0010 ± 0.0002	5.69	0.002	0.73	23	23	2	43
	15	5	0.0041 ± 0.0003	15.35	0.00002	0.97	17	16	1	36
		9	0.0045 ± 0.0004	10.06	0.0002	0.93	22	22	2	42
		12	0.0035 ± 0.0003	12.54	0.00006	0.95	18	18	1	38
	25	5	0.018 ± 0.001	13.89	0.00003	0.98	21	21	4	43
		9	0.019 ± 0.003	7.29	0.001	0.93	29	29	10	50
		12	0.013 ± 0.001	14.30	0.00003	0.98	21	21	2	41
	35	5	0.048 ± 0.005	10.36	0.0001	0.96	25	25	11	45
		9	0.049 ± 0.005	9.00	0.0003	0.95	26	26	10	47
		12	0.041 ± 0.005	8.03	0.0005	0.93	28	28	12	50

Table S5: Summary of Arrhenius fit parameters for different PHBHHx variants. Activation energies (E_a) were determined by fitting the Arrhenius rate law to the temperature-dependent pseudo-first order biodegradation rate constants determined by model fits. The lag phase L was included if the p-value < 0.05 for all temperatures investigated in each soil for each PHBHHx variant. The E_a values determined for the three PHBHHx variants were within uncertainty (95% confidence intervals) for a given soil, supporting that there was no effect of the 3-hydroxyhexanoate content on biodegradation for the current experiments. The R^2 values show that the Arrhenius rate law fit was best for biodegradation data in LUFA 2.2 soil, with fit quality being lower for biodegradation in soils LUFA 6S and 2.4. The lower R^2 values are reflective of sigmoidal profiles between inverse temperature and ln(k), as opposed to linear dependencies expected based on the Arrhenius rate law. Visualization is provided in Figure S12.

Soil	PHBHHx variant	Activation Energy (E_a, kJ mol^{-1})	R^2
LUFA 6S	5	53 ± 28	0.63
	9	63 ± 12	0.83
	12	51 ± 10	0.93
LUFA 2.4	5	78 ± 18	0.79
	9	53 ± 10	0.91
	12	77 ± 24	0.69
LUFA 2.2	5	114 ± 2	1.00
	9	117 ± 4	0.99
	12	107 ± 5	0.98

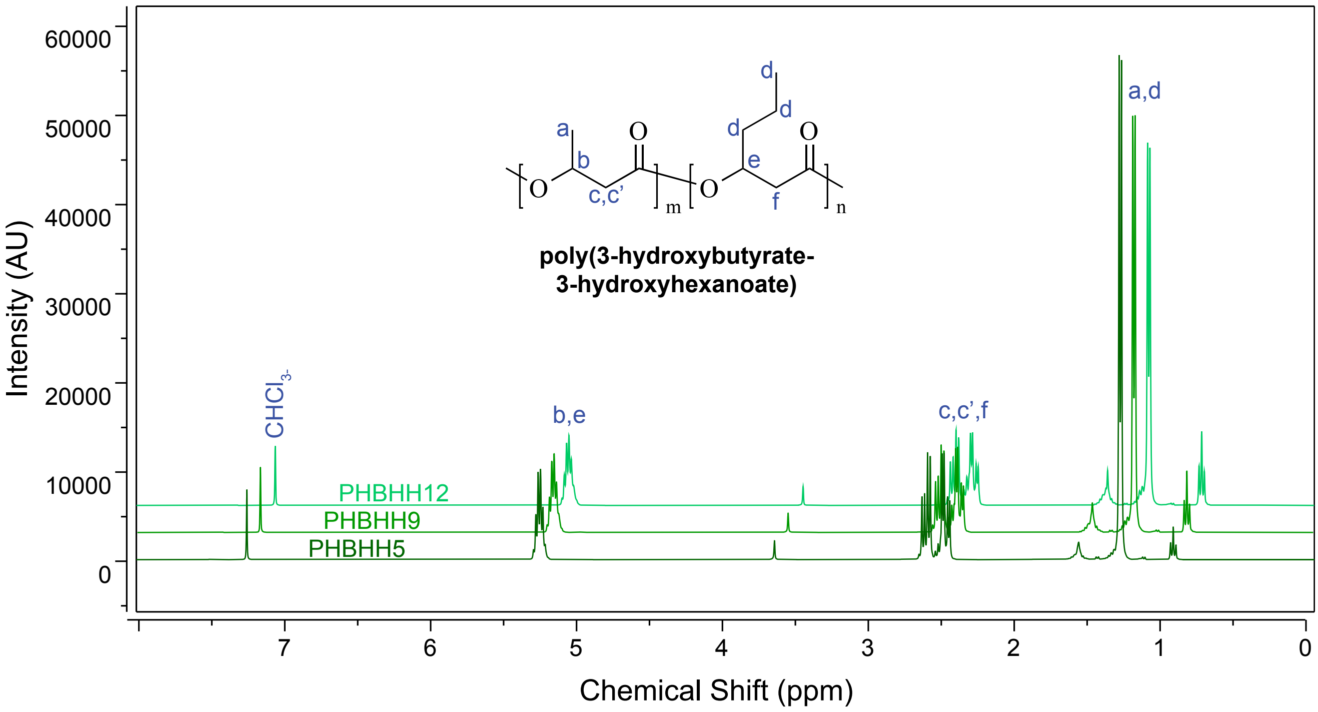

Figure S1: Example PHBHHx ^{1}H NMR spectra. Annotated spectra showing PHBHHx (i.e., PHBHH5, PHBHH9, and PHBHH12 with 5, 9, and 12 % of 3-hydroxyhexanoate (3-HH)) as pure polymer dissolved in deuterated chloroform.

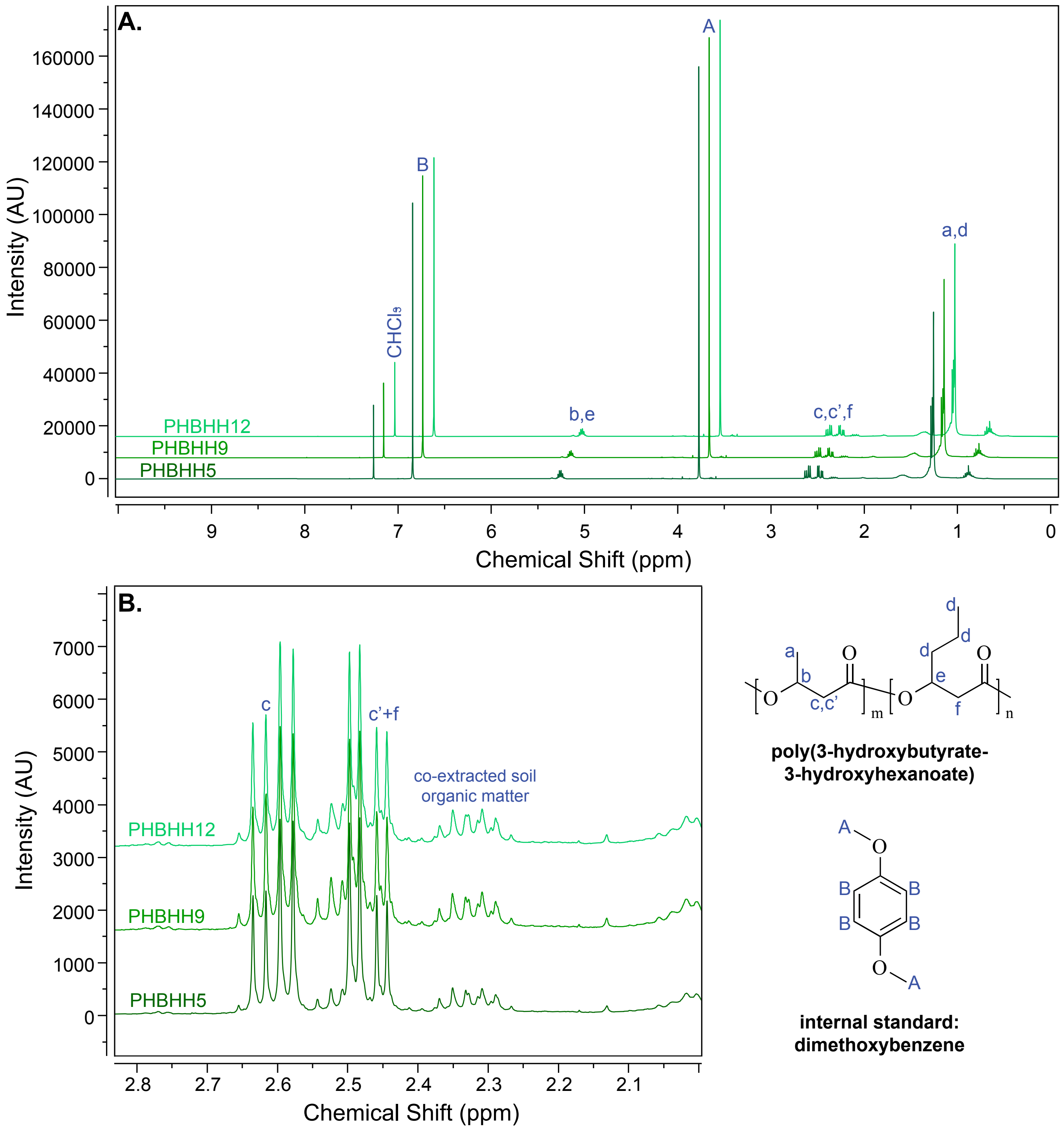

Figure S2: Example PHBHHx ^{1}H NMR spectra with LUFA 6S background. A. Full annotated spectrum showing PHBHHx (i.e., PHBHH5, PHBHH9, and PHBHH12 with 5, 9, and 12 % of 3-hydroxyhexanoate (3-HH)), and the internal standard 1,4-dimethoxybenzene (DMB). B. Inset of PHBHHx region used for PHBHHx quantitation, including determination of the 3-HH molar percentage: the peak to the left corresponds to only the c proton of the 3-hydroxybutyrate monomeric unit while the peak to the right corresponds to both the c' proton of the 3-hydroxybutyrate monomeric unit and the two f protons of the 3-hydroxyhexanoate monomeric unit. The polymer samples were extracted from LUFA 6S soil. Co-extracted soil organic matter does not interfere in the chemical shift region used for PHBHHx quantitation.

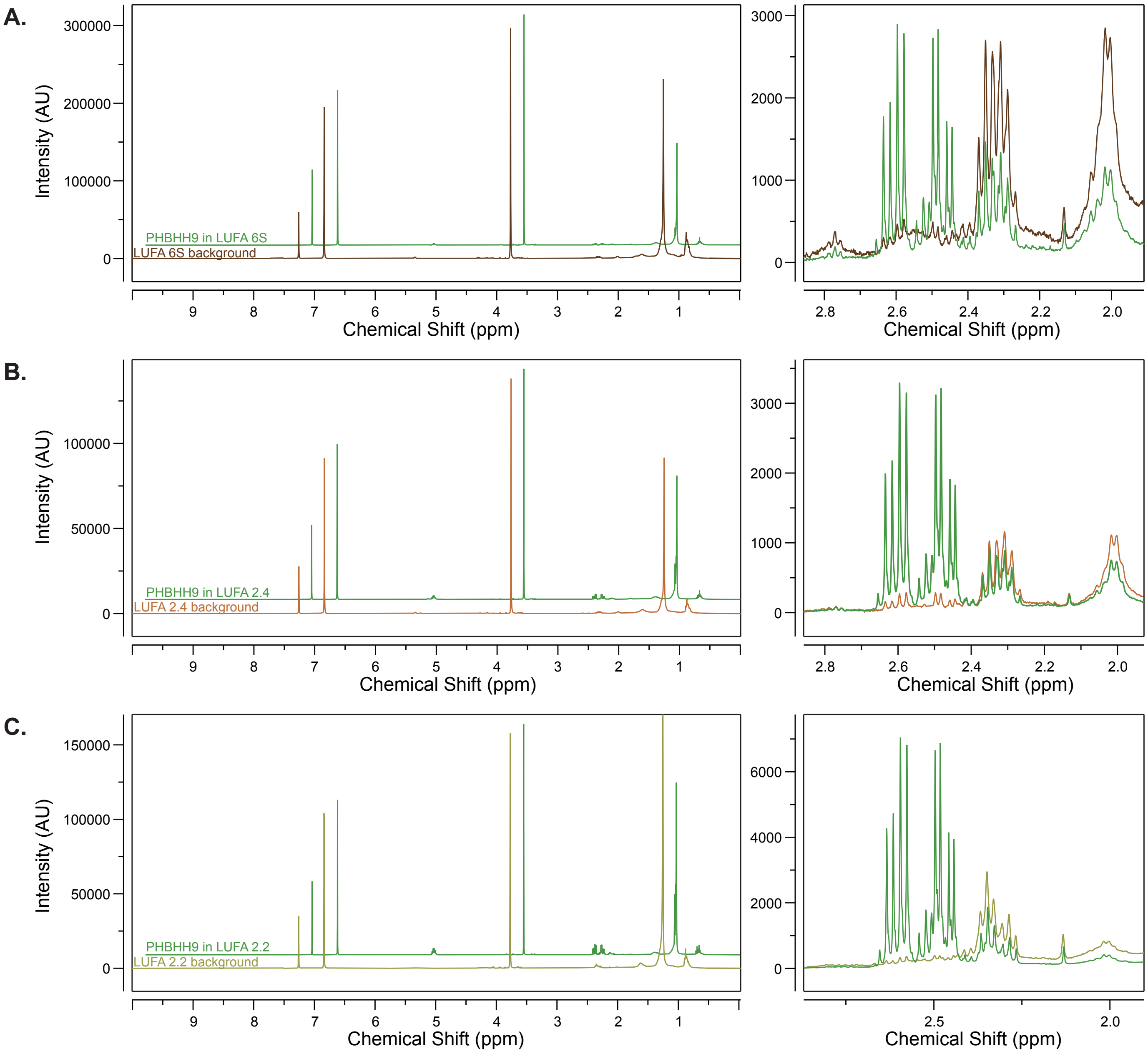

Figure S3: Example soil background in ^{1}H NMR spectra with side panels at the right highlighting the region used for quantification. Note that the left panels are offset to distinguish between background and PHBHHx. A. Residual PHBHH9 in LUFA 6S soil at 25 ºC (green line) compared to LUFA 6S soil background (brown line). B. Residual PHBHH9 in LUFA 2.4 soil at 25 ºC (green line) compared to LUFA 2.4 soil background (brown line). C. Residual PHBHH9 in LUFA 2.2 soil at 25 ºC (green line) compared to LUFA 2.2 soil background (brown line). It is expected that monomers and oligomers would appear upfield from the peaks of interest but were not observed in the extracts generated.

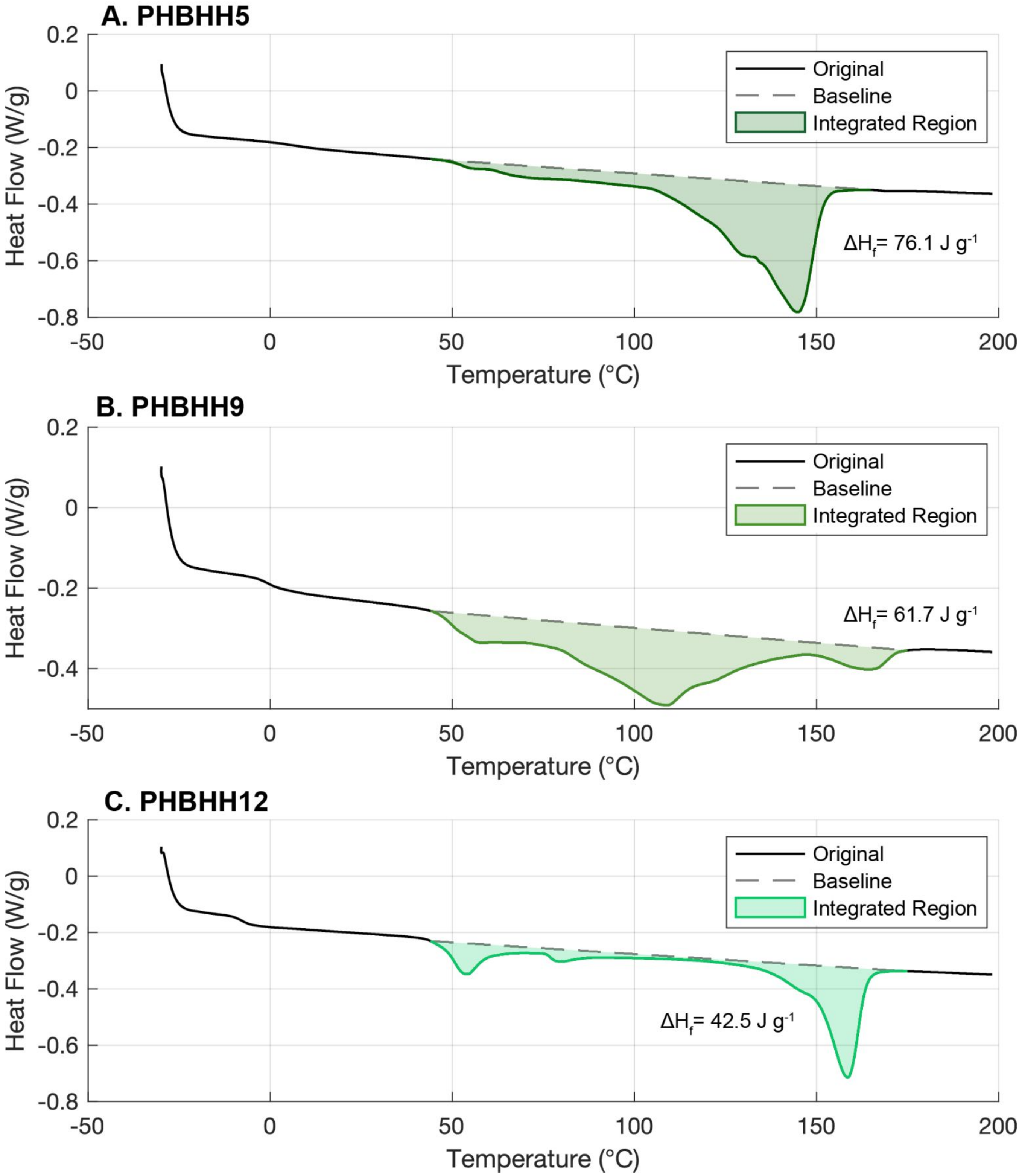

Figure S4: Differential scanning calorimetry (DSC) scans. DSC was performed on PHBHH5 (A), PHBHH9 (B), and PHBHH12 (C). The first melt scans show broad melting behavior that is not uncommon for polyhydroxyalkanoates.The enthalpies of fusion (ΔH_f) were determined and used to calculate the crystallinities of the PHBHHx powders that were used in the incubations (Table S1) using a literature value for ΔH_f of pure poly(3-hydroxybutyrate) of 146 J/g.[6]

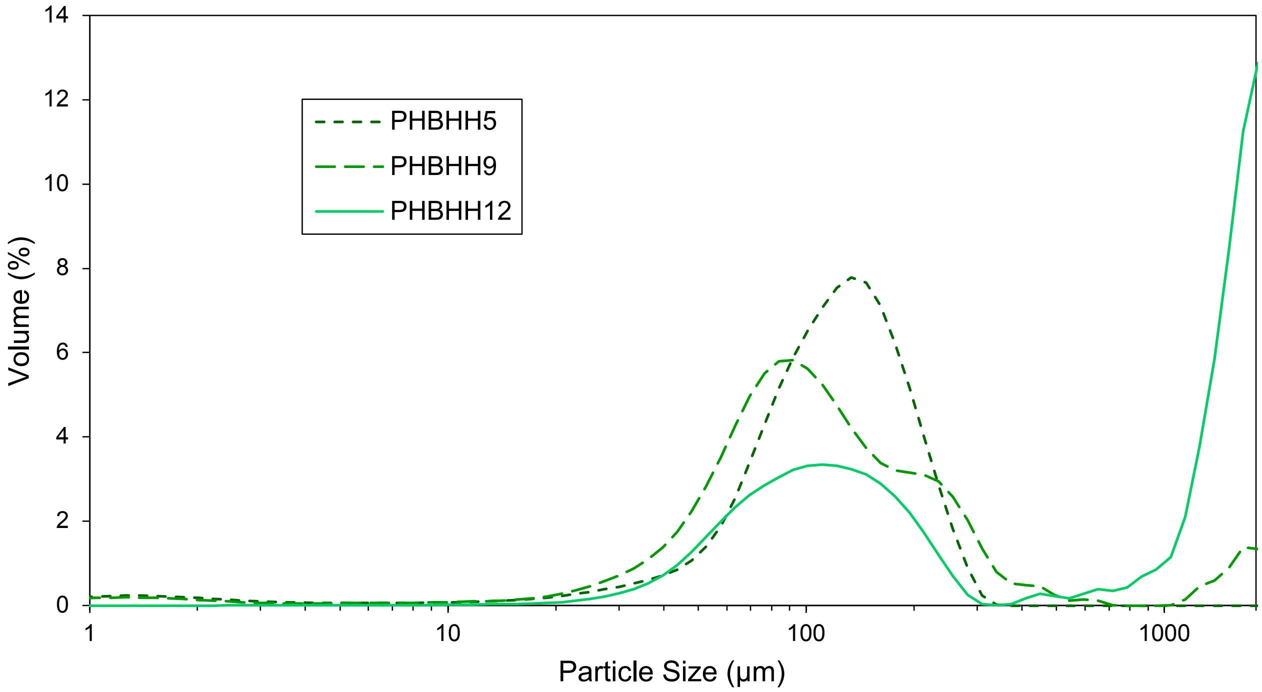

Figure S5: PHBHHx particle size distribution. Particle size distribution for the three poly-3-hydroxybutyrate-3-hydroxyhexanoate (PHBHH5, PHBHH9, and PHBHH12) variant powders used in mass loss experiments in this study analyzed by laser diffraction particle size analysis (LS 13 320, Beckman Coulter, Universal liquid module). The variants PHBHH5, PHBHH9, and PHBHH12 contained 5, 9 and 12 % 3-hydroxyhexanoate, respectively. Generally, the particles for each variant center around 100 µm, but differences are observed between the co-polymers. Namely, larger particles (~1000 µm) are observed for PHBHH9 and PHBHH12 and a bimodal distribution is observed for PHBHH9. The larger particles are suspected to be aggregates that formed in suspension. The differences in the size distribution around 100 µm are believed to be sufficiently small to not cause substantial size-dependency in the biodegradation of the different PHBHHx variants.

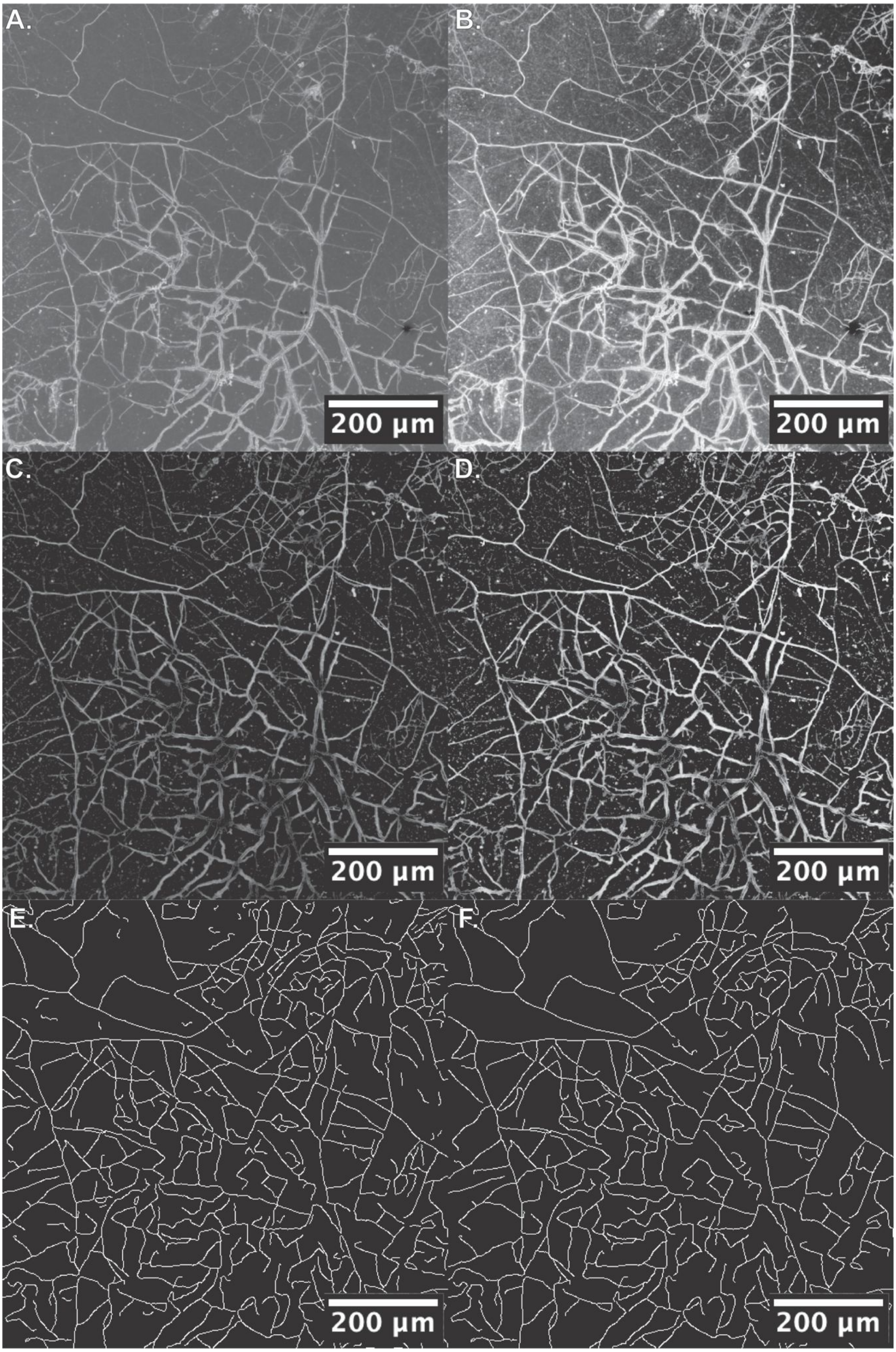

Figure S6: Illustration of the image analysis for hyphal length. A. The initial small tile image, B. after contrast enhancement, C. after background subtraction, D. after the

second contrast enhancement, E. after the ridge detection algorithm was applied, and F. after particle size filter was applied.

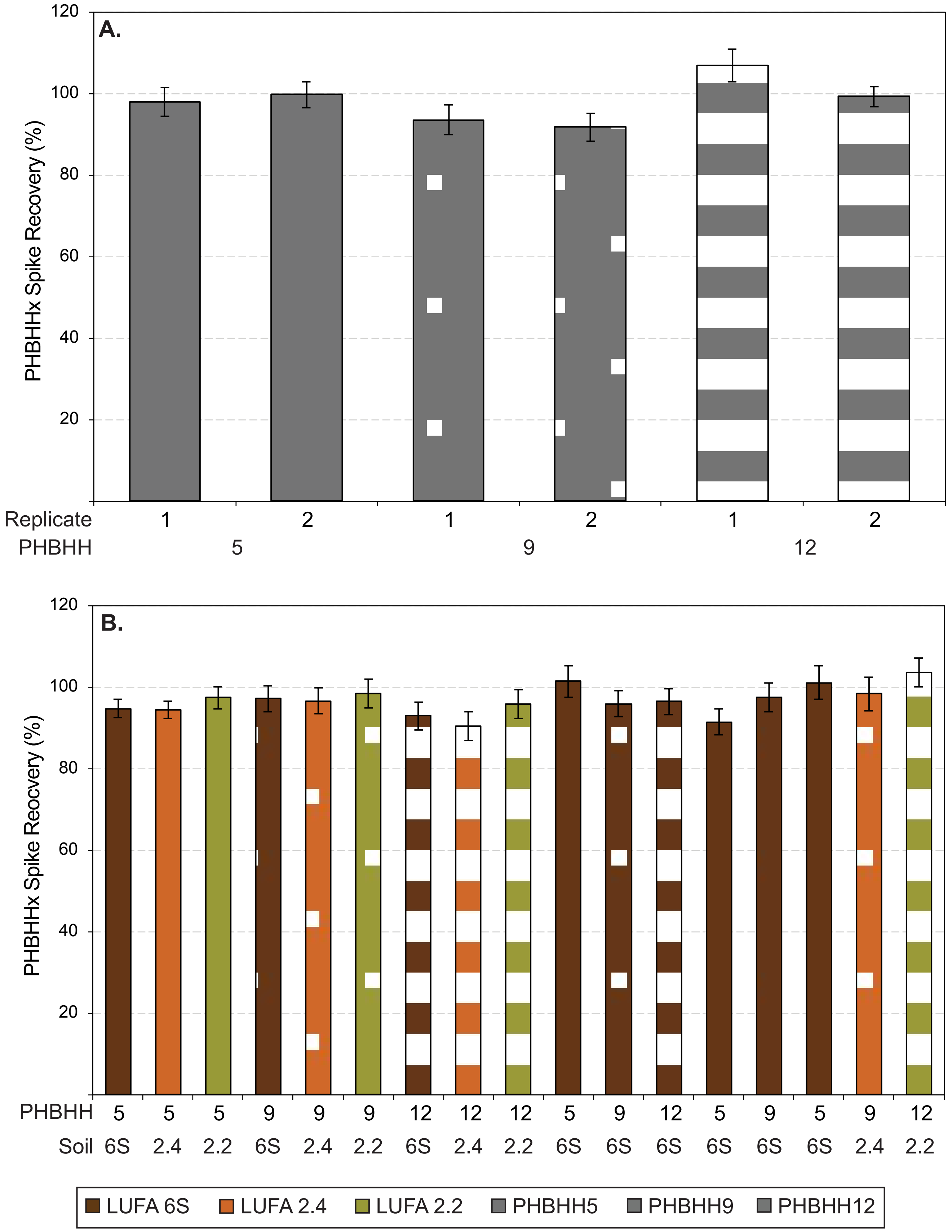

Figure S7: Spike recovery PHBHHx quality control data. Full recoveries of residual PHBHHx from soils was demonstrated by performing PHBHHx (powder) spike recovery experiments in the soils used in this study. Error bars represent propagation

of error of the spiked PHBHHx masses. A. Spike recovery of PHBHHx samples without soil, which confirms no significant losses of PHBHHx to the Soxhlet extraction apparatus and subsequent reconstitution for NMR analysis. B. Spike recovery experiments of PHBHHx variants to soils performed between February 2022 and December 2022, which show that efficient recovery was obtained from the three tested soils over the dates in which experimental samples were also extracted. The average recoveries spiked to soils was 97± 4% for PHBHH5, 97± 1% for PHBHH9, and 97 ± 5% for PHBHH12. Overall, average recovery of PHBHHx was 97 ± 3%. Because the recoveries were indistinguishable from 100% based on propagation of error in measured masses of PHBHHx, no recovery correction factor was used for the quantification of residual PHBHHx in the biodegradation experiments.

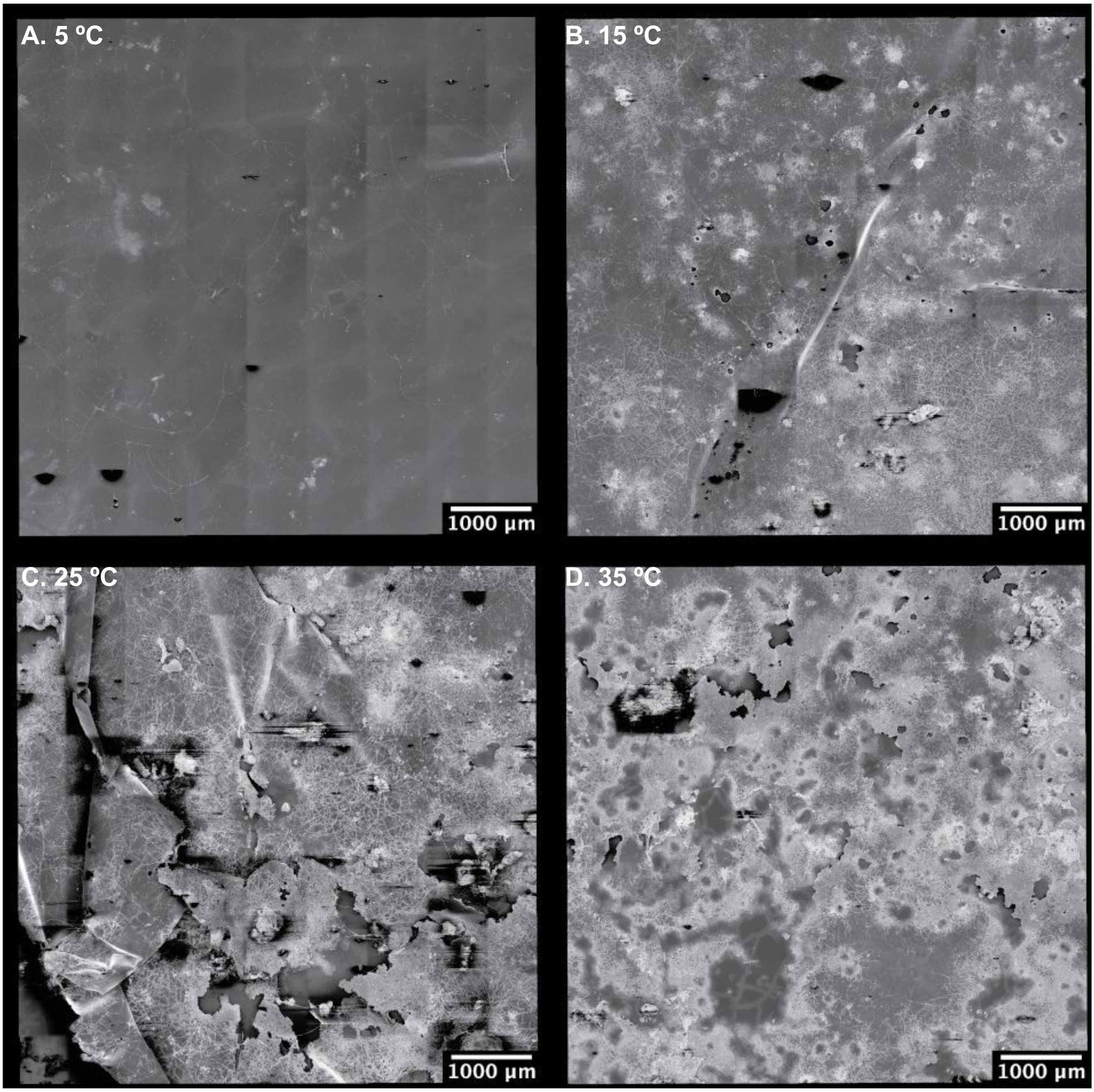

Figure S8: Mosaic image of PHBHH9 films incubated in LUFA 6S soil. A. Sporadic growth of hyphae is observed across the film incubated at 5 °C. B. Formation of hyphal colonies and holes in the PHBHH9 film incubated at 15 °C. C. More extensive colony and hole formation on the PHBHH9 film incubated at 25 °C. D. The PHBHH9 film incubated at 35 °C is nearly fully covered by hyphae and contains a substantial number of holes. Note that the black spots and striated lines originate from surface charging, a well-known phenomenon in SEM imaging.

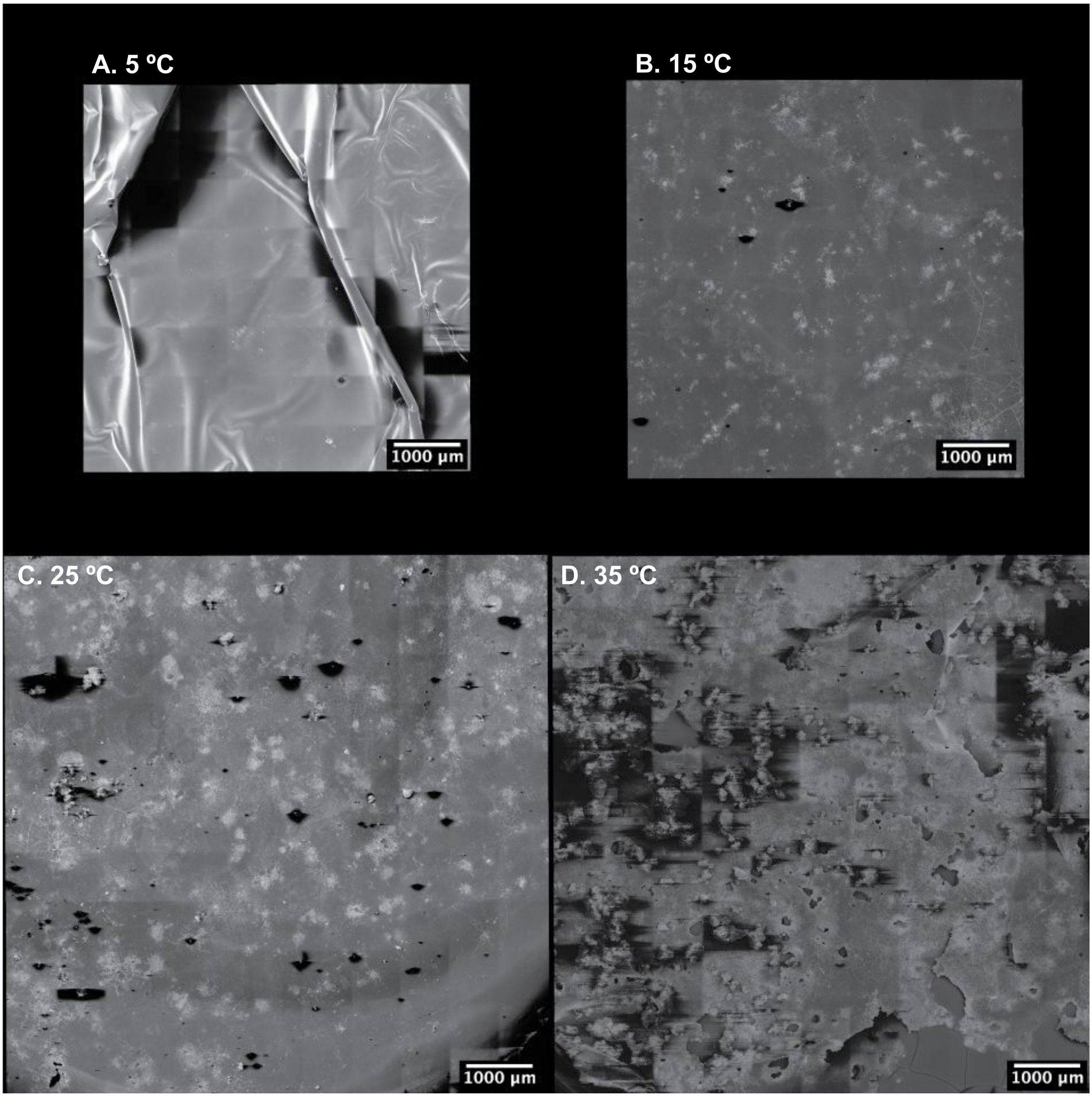

Figure S9: Mosaic image of PHBHH9 films incubated in LUFA 2.4 soil. A. Very little sporadic growth of hyphae is observed across the film incubated 5 °C. B. Formation of hyphal colonies and holes in the PHBHH9 film incubated at 15 °C. C. More extensive hyphal growth, colony, and hole formation on the PHBHH9 film incubated at 25 °C. D. The PHBHH9 film incubated at 35 °C is nearly fully covered by hyphae and contains a substantial number of holes. Note that the black spots and striated lines originate from surface charging, a well-known phenomenon in SEM imaging.

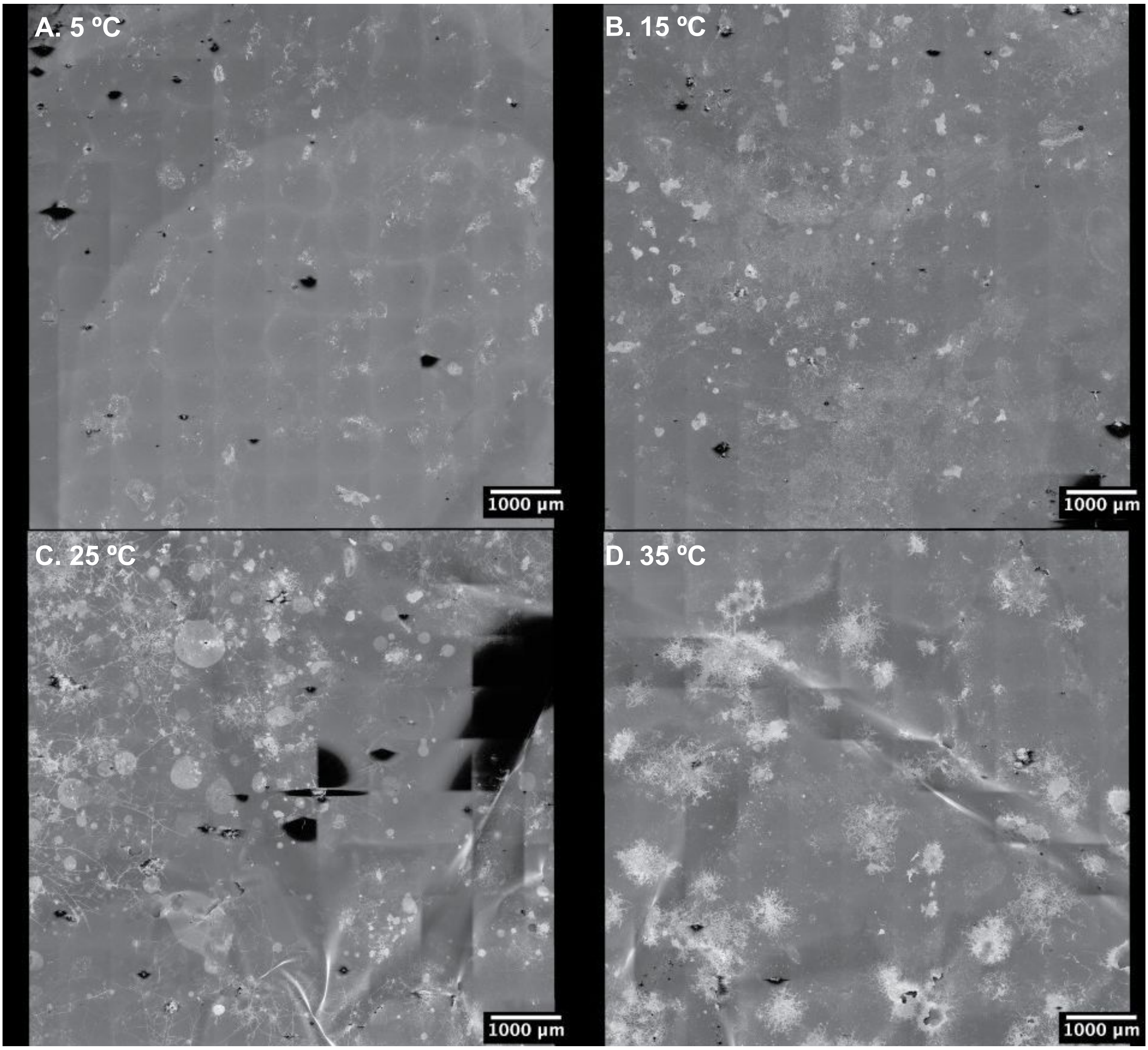

Figure S10: Mosaic image of PHBHH9 films incubated in LUFA 2.2 soil. A. Little hyphae growth is observed across the film incubated at 5 °C. B. Hyphae growth is observed across the film surface and soil particles are adhered to the PHBHH9 film incubated at 15 °C. C. More extensive colony formation is observed on the PHBHH9 film incubated at 25 °C. D. The PHBHH9 film incubated at 35 °C had dense colonies formed on the surface. Note that the black spots and striated lines originate from surface charging, a well-known phenomenon in SEM imaging.

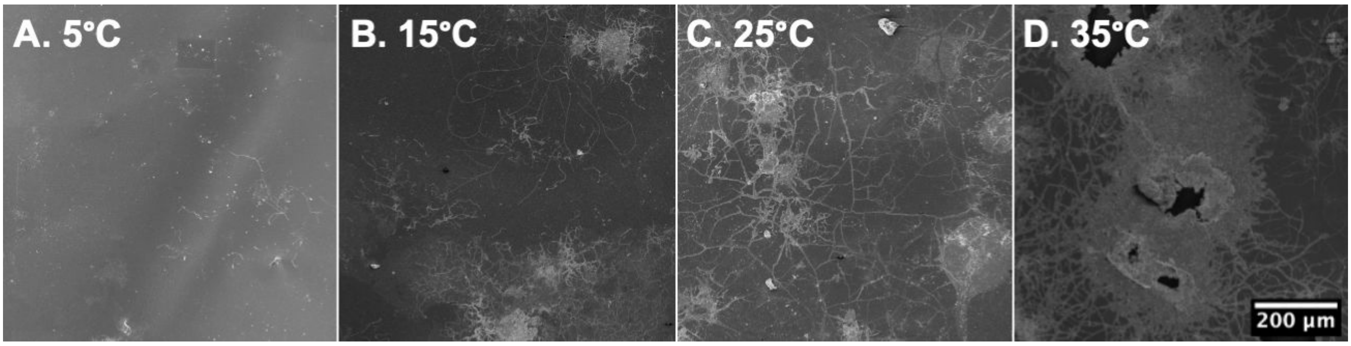

Figure S11: Examples of individual images for PHBHH9 films incubated in LUFA 2.4 soil. The PHBHH9 films (9% 3-hydroxyhexanoate) were incubated for 18 days at 5 °C (A), 15 °C (B), 25 °C (C), and 35 °C (D). The films incubated in LUFA 2.4 soil were more extensively colonized and (bio)degraded than those incubated in LUFA 2.2 but less so than in LUFA 6S (shown in main text, Figure 1).

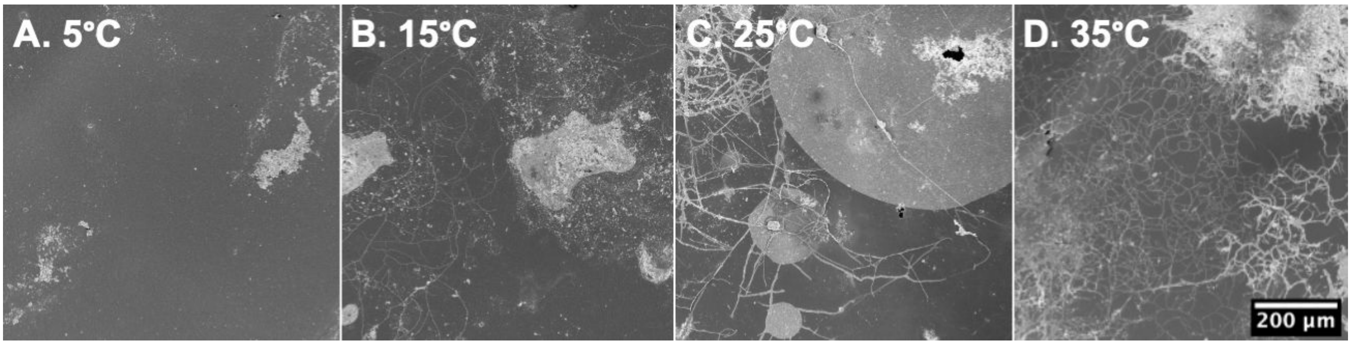

Figure S12: Examples of individual images for PHBHH9 films incubated in LUFA 2.2 soil. PHBHH9 films were incubated for 18 days at 5 (A), 15 (B), 25 (C), and 35 °C (D). Increasing microbial colonization and film deterioration is observed with increasing temperature. In general, the films incubated in the LUFA 2.2 soil were more intact and less biodegraded than films incubated in the other two tested soils.

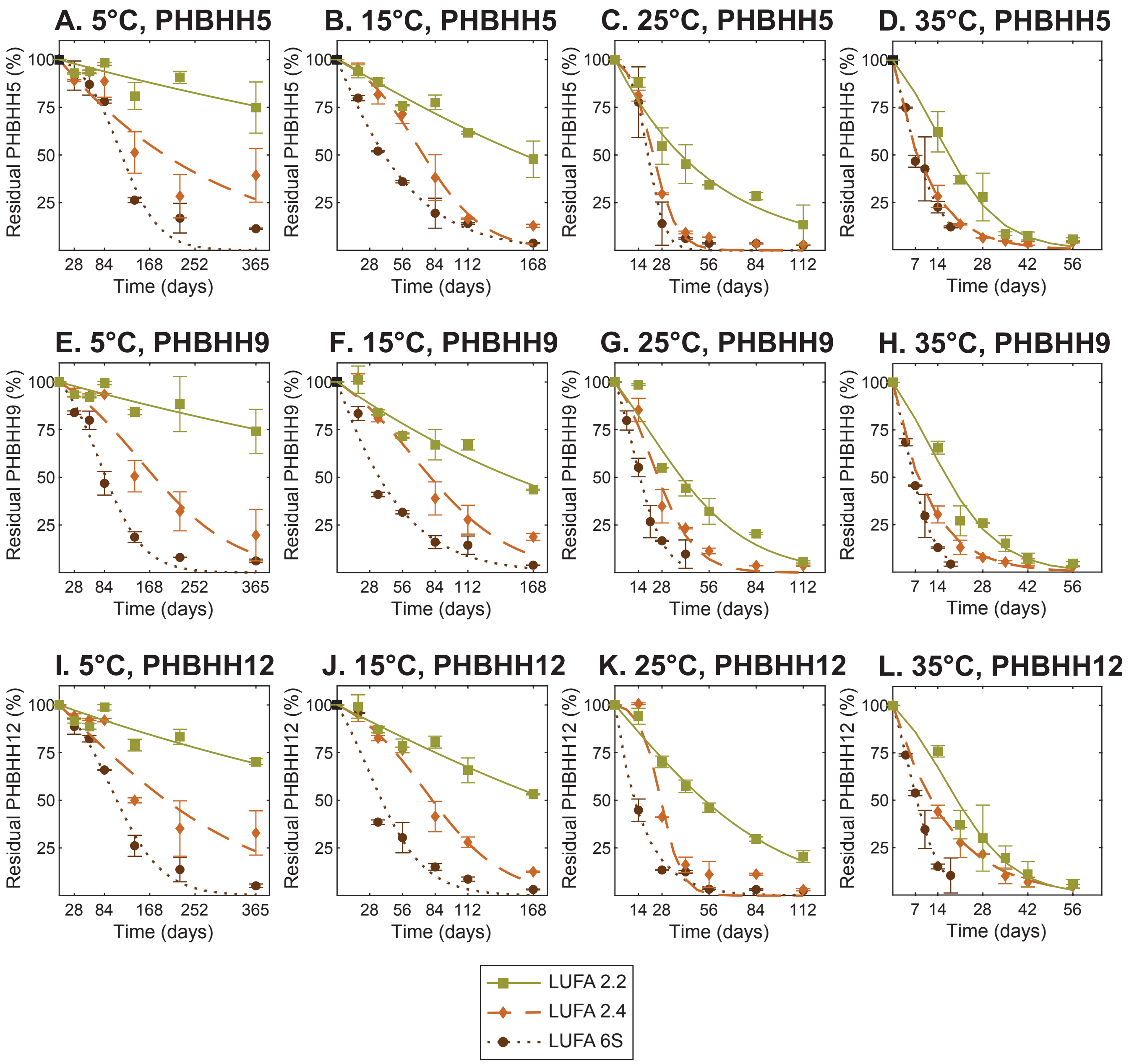

Figure S13: Effect of soil on PHBHHx biodegradation. Re-plotting of biodegradation data to aid in assessing the soil-dependence of biodegradation: LUFA 6S (brown circles), LUFA 2.4 (orange diamonds), and LUFA 2.2 (dark yellow squares) for each 3-hydroxyhexanoate percentage (top row = PHBHH5, middle row = PHBHH9, and bottom row = PHBHH12) and at each temperature (first column = 5 °C, second column = 15 °C, third column = 25 °C, and fourth column = 35 °C).

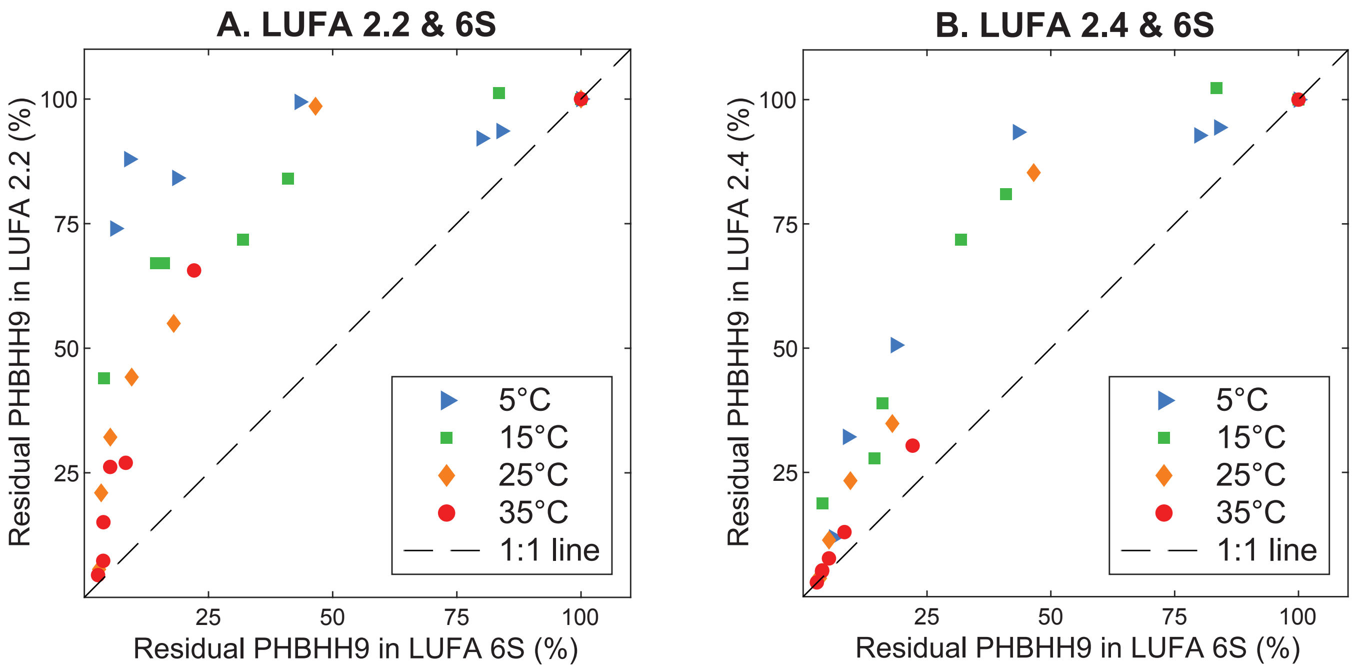

Figure S14: Comparison of biodegradation of PHBHHx among LUFA 2.2 and LUFA 2.4 soils relative to LUFA 6S soil. A. Comparison of biodegradation data in LUFA 2.2 and LUFA 6S soils where each data point corresponds to a sampling time point that was identical for all three soils at a given temperature. Biodegradation appeared slower in LUFA 2.2 compared to LUFA 6S. B. Compares LUFA 2.4 and LUFA 6S soils where each data point corresponds to a sampling time point that was identical for all three soils at a given temperature. Biodegradation in LUFA 2.4 is comparable to LUFA 6S at higher temperatures, but slower at lower temperatures.

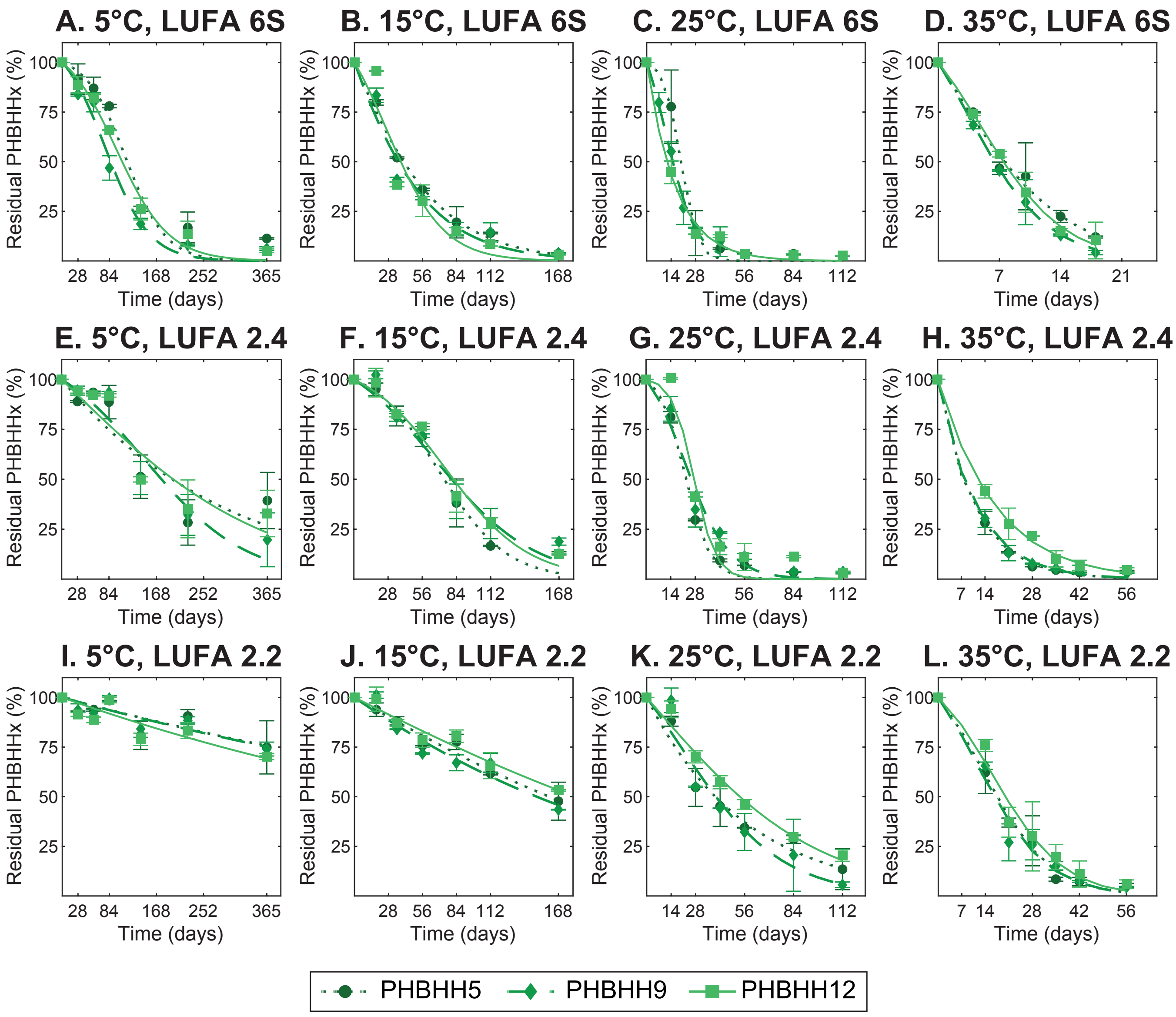

Figure S15: Effect of 3-hydroxyhexanoate percentage on PHBHHx biodegradation. Visualization of the data to focus on the impact of soil on biodegradation profiles: PHBHH5 is represented in dark green circles, PHBHH9 is represented by green diamonds and PHBHH12 is shown as light green squares. Data is shown for all conditions tested: in LUFA 6S soil (top row), LUFA 2.4 soil (middle row), and LUFA 2.2 soil (bottom row); and at each temperature (first column = 5, second column = 15, third column = 25, and fourth column = 35 °C).

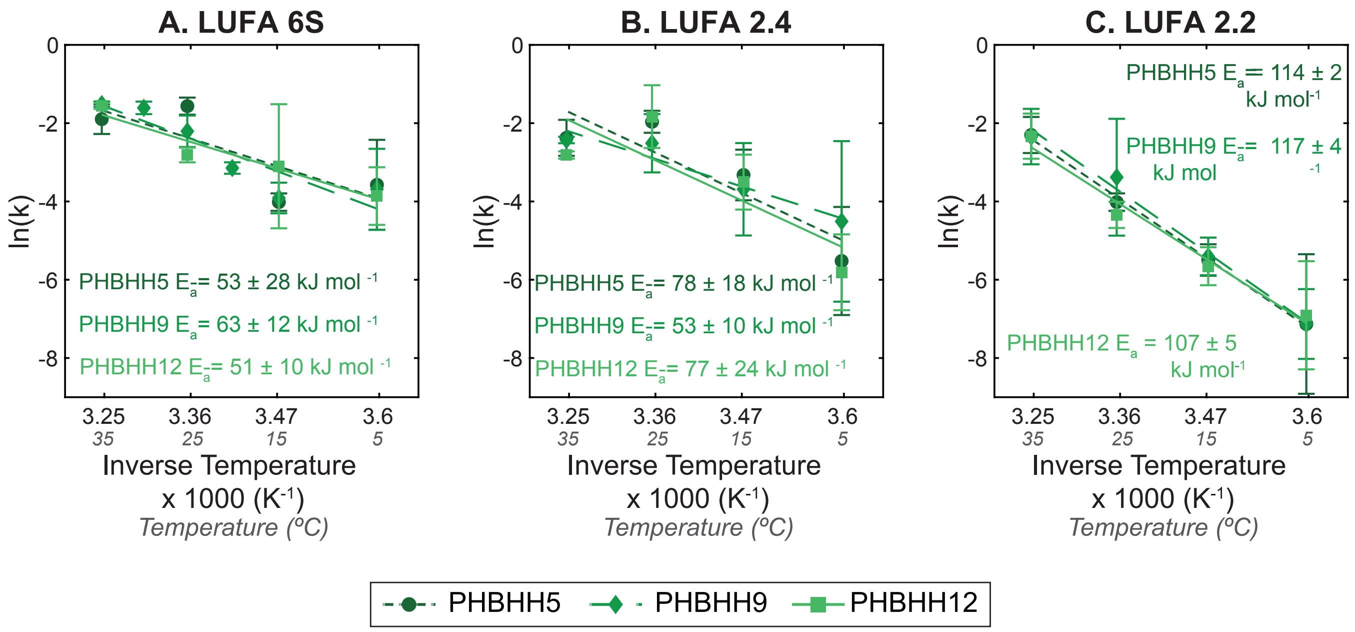

Figure S16: Arrhenius activation energies for three PHBHHx variants. Visualization of fits of the Arrhenius raw law model to the biodegradation data for each of the three tested poly(3-hydroxybutyrate-3-hydroxyhexanoate) (PHBHHx) variants: 5% 3-hydroxyhexanoate (PHBHH5), 9% 3-hydroxyhexanoate (PHBHH9), and 12% 3-hydroxyhexanoate (PHBHH12). The Arrhenius rate law fits support comparable biodegradation dynamics of the three tested variants for any given soil and temperature. The fitted activation energies (E_a) are also summarized in Table S5 along with parameters describing the quality of the model fits (i.e., R^2 values).

Text S3: Image analysis details.

A. Downsizing

We used the subsequent macro code to downsize all tiles of an SEM image saved in one folder. The command for downsizing is in bold letters.

downsizing_macro.ijm

```
#@ File (label = "Input directory", style = "directory") input
#@ File (label = "Output directory", style = "directory") output
#@ String (label = "File suffix", value = ".tif") suffix
processFolder(input);
// function to scan folders/subfolders/files to find files with correct suffix
function processFolder(input) {
	list = getFileList(input);
	list = Array.sort(list);
	for (i = 0; i < list.length; i++) {
		if(File.isDirectory(input + File.separator + list[i]))
			processFolder(input + File.separator + list[i]);
		if(endsWith(list[i], suffix))
			processFile(input, output, list[i]);
	}
}

function processFile(input, output, file) {
	// open image tile
	open(input + File.separator + file);

	// downsizing image tile
	run("Size...", "width=510 height=512 depth=1 constrain average
interpolation=Bilinear");
// save downsized image tile
save(output + File.separator + file);
		print("Processing: " + input + File.separator + file);
		print("Saving to: " + output);

}

run("Close All");
```

B. Stitching of images

Either the SE2 or the InLens channel was stitched first by using Fiji's "Grid/Collection stitching" functionality with the settings "filename defined position", 10% tile overlap, linear blending and "compute overlap". This results in the stitched image and a text file containing the computed exact tile positions. This text file was thereafter used to stitch the second channel with the same tile positions. (Stitching settings: "position from file", "defined by TileConfiguration", linear blending)

C. Hyphae detection

The hyphae were detected and quantified by applying the hyphae detection macro to the InLens images with the following settings: Line width = 2, High contrast = 600, Low contrast = 0, Minimum line length = 7, Minimum area = 40, Approx. slice size = 500. In case the algorithm failed, the slice size was adjusted accordingly.

hyphae_detection_macro.ijm

```
#@ int(label="Line width") line_width
#@ int(label="High contrast") high_contrast
#@ int(label="Low contrast") low_contrast
#@ int(label="Minimum line length") minimum_line_length
```

```
#@ int(label="Minimum area (pixel)") minimum_area
#@ File (label = "Input image file", style = "file") path
#@ File (label = "Output directory", style = "directory") output
#@ int(label="Approx. slice size") slice_size

// check if tmp is existing =====================================================
// make an error if there is already a tmp directory

tmp_dir = File.isDirectory(output + File.separator + "tmp");
if( tmp_dir == 1){
   exit(output + File.separator + "tmp directory already exists. Please delete it before running this
macro.");
}

// open original image ==============================================
open(path);
selectImage(File.getName(path));
title = getTitle();

//get pixel properties
getPixelSize(unit, pixelWidth, pixelHeight);

// pre ridge detection filtering
=============================================================

run("Enhance Contrast...", "saturated=0 normalize equalize");

// background substraction + deletion
run("Duplicate...", "title=background.tif");
run("Remove Outliers...", "radius=10 threshold=10 which=Bright");
imageCalculator("Subtract create", title,"background.tif");

//close not used images
selectImage(title);
close();
selectImage("background.tif");
close();

//again enhance contrast
selectImage("Result of "+ title);
run("Enhance Contrast...", "saturated=0 normalize equalize");
rename(title);

// slice image ====================================================

print("slicing image");

//get id and dimension
id = getImageID();
title = getTitle();

width = getWidth();
height = getHeight();

// save slices as tiles in tmp directory
File.makeDirectory(output + File.separator + "tmp");

// compute number of slices necessary
n_x = Math.ceil(width/slice_size);
n_y = Math.ceil(height/slice_size);
```

```
tileWidth = Math.ceil(width/n_x);
tileHeight = Math.ceil(height/n_y);

//add black pixels to the right and bottom side to prevent rounding errors
// its necessary to add the old image as an overlay onto a black image with the final size
newImage("black_background", "8-bit black", n_x*tileWidth, n_y*tileHeight, 1);
selectImage("black_background");
run("Add Image...", "image="+title+" x=0 y=0 opacity=100");
selectImage(title);
close();
selectImage("black_background");
run("Flatten");
run("8-bit");
rename(title);
selectImage("black_background");
close();

// set pixel properties
Stack.setXUnit(unit);
Stack.setYUnit(unit);
run("Properties...", "channels=1 slices=1 frames=1 pixel_width="+pixelWidth+"
pixel_height="+pixelHeight+" voxel_depth=1");

//get id and dimension
selectImage(title);
id = getImageID();

width = getWidth();
height = getHeight();

// Chop an image into tiles
getLocationAndSize(locX, locY, sizeW, sizeH);
for (y = 0; y < n_y; y++) {
offsetY = y * height / n_y;
 for (x = 0; x < n_x; x++) {
offsetX = x * width / n_x;
selectImage(id);
 call("ij.gui.ImageWindow.setNextLocation", locX + offsetX, locY + offsetY);
tileTitle = title + " [" + (y+1) + "," + (x+1) + "]";
 run("Duplicate...", "title=" + tileTitle);
makeRectangle(offsetX, offsetY, tileWidth, tileHeight);
 run("Crop");
 save(output + File.separator + "tmp" + File.separator + "slice_" + n_x + "x" + n_y + "_" +
((x+1)+y*n_x) + ".tif");
 close();
}
}
selectImage(id);
close();


// run ridge detection
=================================================================

print("running ridge detection");

// import image sequence
File.openSequence(output + File.separator + "tmp");
```

```
title = getTitle();

// run ridge detection
run("Ridge Detection", "line_width="+ line_width +" high_contrast=" + high_contrast +
" low_contrast="+ low_contrast +" minimum_line_length="+ minimum_line_length +" maximum=0
extend_line make_binary " +
"method_for_overlap_resolution=NONE stack");

selectImage(title);
close();

// filter particles on binary image ================================================
print("filtering binary image");

selectImage(title +" Detected segments");

run("Analyze Particles...", "size="+ minimum_area +"-Infinity pixel show=Masks stack");
run("Invert LUT");
rename(title);

selectImage(title +" Detected segments");
close();

// second ridge detection on binary image ===========================================
print("running second ridge detection");

selectImage(title);

// run ridge detection
run("Ridge Detection", "line_width=1 high_contrast=600" +
" low_contrast=0 minimum_line_length=3 maximum=0 extend_line displayresults make_binary " +
"method_for_overlap_resolution=NONE stack");

close("Junctions");


close("Results");

// calculate total length and write to file
selectWindow("Summary");
len_array = Table.getColumn("Length");
Array.getStatistics(len_array, minimum, maximum, mean);
sum = mean * lengthOf(len_array);

file=File.open(output + File.separator + "hyphae_length.txt");
print(file, sum);

close("Summary");

// stitch binary image
=================================================================

print("stitch and save binary image");

// empty tmp directory
tmp_dir = output + File.separator + "tmp" ;
fileList = getFileList(tmp_dir);
print(fileList.length);
for(i=0; i<fileList.length; i++) {
        File.delete(tmp_dir + File.separator + fileList[i]);
```

```
}

// save binary as tiles in tmp directory

selectImage(title +" Detected segments");
run("Image Sequence... ", "select=[" + output + File.separator + "tmp] dir=[" + output + File.separator
+ "tmp" + File.separator + "] format=TIFF name=binary_ start=1 digits=3");
close();


// stitch binary image
run("Grid/Collection stitching", "type=[Grid: row-by-row] order=[Right & Down                ] " +
"grid_size_x="+ n_x +" grid_size_y="+ n_y +" tile_overlap=0 first_file_index_i=1 directory=[" + output
+ File.separator + "tmp] " +
"file_names=binary_.tif output_textfile_name=TileConfiguration.txt fusion_method=[Linear Blending] "
+
"regression_threshold=0.30 max/avg_displacement_threshold=2.50
absolute_displacement_threshold=3.50 " +
"computation_parameters=[Save memory (but be slower)] image_output=[Fuse and display] use");

// set pixel properties
Stack.setXUnit(unit);
Stack.setYUnit(unit);
run("Properties...", "channels=1 slices=1 frames=1 pixel_width="+pixelWidth+"
pixel_height="+pixelHeight+" voxel_depth=1");

saveAs("Tiff", output + File.separator + "Binary.tif");
close();

// stitch overlay image
============================================================

print("stitch and save overlay image");

// empty tmp directory
fileList = getFileList(tmp_dir);
for(i=0; i<fileList.length; i++) {
	File.delete(tmp_dir + File.separator + fileList[i]);
}

// save overlay as tiles in tmp directory
File.makeDirectory(output + File.separator + "tmp");

selectImage(title);
run("Image Sequence... ", "select=[" + output + File.separator + "tmp] dir=[" + output + File.separator
+ "tmp"+File.separator+"] format=TIFF name=overlay_ start=1 digits=3");
close();


//export overlay as ROIset for each tile

fileList = getFileList(output + File.separator + "tmp");

for(i=0; i<fileList.length; i++) {
	open(output + File.separator + "tmp" + File.separator + fileList[i]);
	selectImage(fileList[i]);
	overlay_info = getInfo("overlay");
	if (overlay_info.length>0){
		run("To ROI Manager");
```

```
			roiManager("Save", output +  File.separator + "tmp" + File.separator + "RoiSet_" +
substring(fileList[i], 0, indexOf(fileList[i], ".")) +".zip");
			roiManager("Delete");
		}
		close(fileList[i]);
}


close("ROI Manager");

// stitch images
run("Grid/Collection stitching", "type=[Grid: row-by-row] order=[Right & Down                ] " +
"grid_size_x="+n_x+" grid_size_y="+n_y+" tile_overlap=0 first_file_index_i=1 directory=["+output+
File.separator + "tmp]  " +
"file_names=overlay_{iii}.tif output_textfile_name=TileConfiguration.txt fusion_method=[Linear
Blending]  " +
"regression_threshold=0.30 max/avg_displacement_threshold=2.50
absolute_displacement_threshold=3.50  " +
"computation_parameters=[Save memory (but be slower)] image_output=[Fuse and display] use");

// set pixel properties
Stack.setXUnit(unit);
Stack.setYUnit(unit);
run("Properties...", "channels=1 slices=1 frames=1 pixel_width="+pixelWidth+"
pixel_height="+pixelHeight+" voxel_depth=1");

// add overlay form RoiSet files---

// read tile configuration file
tile_config = File.openAsString(output + File.separator + "tmp" + File.separator +
"TileConfiguration.txt");

//Separate file into rows
tile_config = split(tile_config,"\n");

for(i=0; i<fileList.length; i++) {

		// get config for certain tile
		tile_config_i = Array.filter(tile_config, fileList[i]);
		tile_config_i = tile_config_i[0];
		tile_config_i = substring(tile_config_i, indexOf(tile_config_i, "(")+1, indexOf(tile_config_i, ")"));

		tile_config_xy = split(tile_config_i, ", ");

		t_x = parseInt(tile_config_xy[0]);
		t_y = parseInt(tile_config_xy[1]);


		//add overlay from RoiSet
		ROI_file = output +  File.separator + "tmp" + File.separator + "RoiSet_" + substring(fileList[i],
0, indexOf(fileList[i], ".")) +".zip";

		if (File.exists(ROI_file)) {
					roiManager("Open", ROI_file);
					RoiManager.translate(t_x, t_y);
					run("From ROI Manager");
					roiManager("Delete");
			}

}
```

```
// save and close windows
run("To ROI Manager");
roiManager("Save", output +  File.separator + "ROI.zip");
run("From ROI Manager");
roiManager("Delete");
close("ROI Manager");
saveAs("Tiff", output +  File.separator + "Overlay.tif");
close();

// delete tmp directory
fileList = getFileList(tmp_dir);
for(i=0; i<fileList.length; i++) {
		File.delete(tmp_dir + File.separator + fileList[i]);
}
File.delete(tmp_dir);

print("Macro completed");
```

References.

(1) Steger, C. An unbiased detector of curvilinear structures. *IEEE Transactions on Pattern Analysis and Machine Intelligence* **1998**, *20* (2), 113-125. DOI: doi:10.1109/34.659930.

(2) Schneider, C. A.; Rasband, W. S.; Eliceiri, K. W. NIH Image to ImageJ: 25 years of image analysis. *Nat Methods* **2012**, *9* (7), 671-675. DOI: 10.1038/nmeth.2089.

(3) Arzt, M.; Deschamps, J.; Schmied, C.; Pietzsch, T.; Schmidt, D.; Tomancak, P.; Haase, R.; Jug, F. LABKIT: Labeling and Segmentation Toolkit for Big Image Data. *Frontiers in Computer Science* **2022**, *4*. DOI: 10.3389/fcomp.2022.777728.

(4) Willcott, M. R. MestRe Nova. *Journal of the American Chemical Society* **2009**, *131* (36), 13180. DOI: doi.org/10.1021/ja906709t.

(5) Alata, H.; Aoyama, T.; Inoue, Y. Effect of Aging on Mechanical Properties of Poly(3-hydroxybutyrate-co-3-hydroxyhexanoate). *Macromolecules* **2007**, *40*, 4546-4551. DOI: 10.1021/ma070418i.

(6) Barham, P. J.; Keller, A.; O'tun, E. L.; Holmes, P. A. Crystallization and morphology of a bacterial thermoplastic: poly-3-hydroxybutyrate. *Journal of Materials Science* **1984**, *19*, 2781-2794.
